# Spatial Organization of Mesenchymal Stem Cells *In Vitro*—Results from a New Individual Cell-Based Model with Podia

**DOI:** 10.1371/journal.pone.0021960

**Published:** 2011-07-08

**Authors:** Martin Hoffmann, Jens-Peer Kuska, Matthias Zscharnack, Markus Loeffler, Joerg Galle

**Affiliations:** 1 Biomathematics and Bioinformatics Group, Department of Knowledge Engineering, Maastricht University, Maastricht, The Netherlands; 2 Interdisciplinary Centre for Bioinformatics, University of Leipzig, Leipzig, Germany; 3 Center for Biotechnology and Biomedicine, University of Leipzig, Leipzig, Germany; 4 Institute for Medical Informatics, Statistics and Epidemiology, University of Leipzig, Leipzig, Germany; Rutgers University, United States of America

## Abstract

Therapeutic application of mesenchymal stem cells (MSC) requires their extensive *in vitro* expansion. MSC in culture typically grow to confluence within a few weeks. They show spindle-shaped fibroblastoid morphology and align to each other in characteristic spatial patterns at high cell density. We present an individual cell-based model (IBM) that is able to quantitatively describe the spatio-temporal organization of MSC in culture. Our model substantially improves on previous models by explicitly representing cell podia and their dynamics. It employs podia-generated forces for cell movement and adjusts cell behavior in response to cell density. At the same time, it is simple enough to simulate thousands of cells with reasonable computational effort. Experimental sheep MSC cultures were monitored under standard conditions. Automated image analysis was used to determine the location and orientation of individual cells. Our simulations quantitatively reproduced the observed growth dynamics and cell-cell alignment assuming cell density-dependent proliferation, migration, and morphology. In addition to cell growth on plain substrates our model captured cell alignment on micro-structured surfaces. We propose a specific surface micro-structure that according to our simulations can substantially enlarge cell culture harvest. The ‘tool box’ of cell migratory behavior newly introduced in this study significantly enhances the bandwidth of IBM. Our approach is capable of accommodating individual cell behavior and collective cell dynamics of a variety of cell types and tissues in computational systems biology.

## Introduction

Over the past decade mesenchymal stem cells (MSC) derived from bone marrow, adipose, and many other tissues have intensively been investigated with respect to their tissue regeneration prospects [1], [2], [3]. MSC have high proliferative potential and capability of differentiating into various cell types [4], [5]. Their therapeutic deployment ranges from supplementing bone marrow transplantations to treatment of various diseases, including osteoarthritis [6] and myocardial infarction [7]. Presently, MSC application in asthma, radiation exposure, and neurological disorders is being explored [8].

Any therapeutic use of MSC relies on their extensive expansion. One major aspect of cell expansion is the selection of highly potent cells from tissue biopsy. Various protocols have been suggested for effectively isolating MSC with high regenerative potential and homogeneity [9], [10], [11]. In addition to soluble factors such as oxygen [12], [13], [14], [15] and growth factors [16], [17], MSC fates have been demonstrated to be controlled by substrate stiffness [18], geometry [19], micro/nano-structure [20], [21] and surface chemistry [22].

These rapid experimental developments are paralleled by striking progress in the mathematical sciences dedicated to the modeling and simulation of tissue formation dynamics. However, only few approaches address MSC organization on the cellular level [14], [23], [24]. Recently, we developed a three-dimensional IBM of MSC culture [14] that provides a consistent explanation for numerous experimental findings on the oxygen dependence of MSC expansion and chondrogenic differentiation *in vitro*.

During cultivation MSC undergo phenotypic changes that enable their early cell shape-based classification [25]. These changes in cell morphology have, however, not been considered in our previous spherical cell model. As a consequence, the spatial cell distribution and cell-cell alignment in dense culture were not appropriately described. Irrespectively, these phenomena appear to substantially affect MSC migration and expansion [26], as well as tissue-specific cell-cell communication, lineage priming, and differentiation [27], [28]. This necessitates the development of improved models of cell migration that account for essential features of MSC shape and behavior.

Models of individual migrating cells have reached a very high level of detail and complexity [29], [30], [31], [32]. Typically, individual cell-based models follow one of two paradigms: either geometric modeling of cells with cell migration according to Brownian-type dynamics (using Langevin equations) [33], [34], [35], [36], or cellular automata-based cell modeling by the Cellular Potts Model (CPM) with cell dynamics being determined according to Monte Carlo steps (using the Metropolis algorithm) [30], [37], [38]. The CPM captivates by its efficiency and direct interoperability with other grid-based methods such as numerical integration of diffusion equations. On the other hand geometric models appear more convenient for inclusion of long range physical forces [28], [39] and an explicit time scale. Spontaneous cell migration on homogeneous substrates has been shown to be approximated by Brownian-like dynamics [40], [41] while a quantitative comparison appears to be still missing for the CPM.

Generalized cell shapes were already introduced by models for Dictyostelium discoideum [42], [43], Myxococcus xanthus [44], [45], blood vessel formation [37], [38], gastrulation [39], [46] and response to cell stretching [47]. For a recent comprehensive review on cell motion see also [48]. The present work makes progress along these lines by expanding our previous spherical cell model [34], [49], [50] by explicitly representing cell podia and their dynamics in a computationally efficient manner. This allows for a more realistic description of single cell migration and, as a consequence, the spatio-temporal organization of whole cell populations - even on micro-structured surfaces. Clearly, our new model adheres to the geometric modeling paradigm, however with Brownian dynamics replaced by podia dynamics, which combine physical (podium elongation) and stochastic (podium generation and inactivation) models. Although podia directional control is strongly influenced by the local geometry and properties of the substrate [51], [52] podia dynamics on homogeneous surfaces are generally thought to be governed by random generation and deletion rates [53], [54]. Apparently, the most extensive results in this area have been obtained for dendrite and growth cone dynamics in neuroscience [55], [56], [57]. Apart from these phenomenological approaches several studies have focused on the molecular basis of podia dynamics [58], [59], [60]. Here, we study the consequences of podia formation for cell migration in dense culture. Our new model is able to reproduce quite a number of experimental results on cell expansion from literature and our own lab. This also includes capturing of cell-cell alignment domains and swirl-like patterns. Similar structures were modeled by discrete and continuous mathematical models [61] and observed in cell-based simulation [45]. In addition to these alignment phenomena, our present model enabled us to predict a substantial increase in cell culture harvest for a starlike surface micro-structure.

## Results

We first introduce the basics of our new IBM and compare experimental and computational results on MSC expansion *in vitro*. MSC growth dynamics and spatial organization are assessed in terms of the number of cells, mean population radius, and cell-cell alignment. We illustrate the general model behavior by presenting simulation results with essential model parameters being varied. Second, we demonstrate our model's capacity to account for contact guidance by micro-structured substrates and predict a micro-structure that according to our simulations can significantly enhance cell yield in *in vitro* cell expansion.

### Model outline

**Figure 1:**
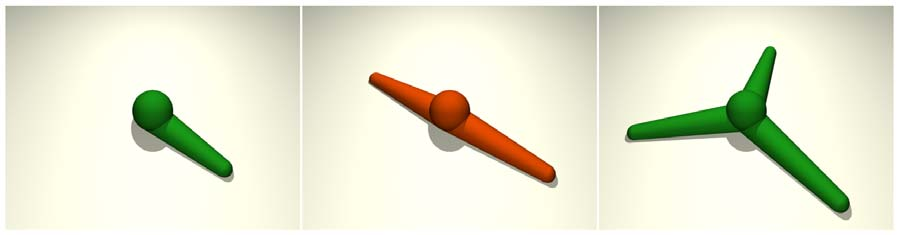
Model cells. Three model cells with 1, 2, and 3 podia, respectively. The podium of the cell with only 1 podium (left) is shorter because of cell movement. The cells with 2 and 3 podia (middle, right) are spread out and resting (see Modeling methods section E).

Our new IBM builds on previous models of Galle and Drasdo [34], [49], [50] representing cells as elastic spheres that can form contacts, move, grow and divide. The cell dynamics are determined by attractive and repulsive interaction forces between cells and between cells and the substrate (Modeling methods section A). This approach is carried over to the present model in that the spherical cell bodies are modeled accordingly. In addition, cells are supplied with podia that generate protrusion and traction forces for cell spreading and movement (Figure 1). Initially, podia of zero length are generated randomly. They subsequently elongate (actin polymerization [58], [59]) while building up a traction force between podium tip and cell body. Cell bodies generally integrate the dragging forces of several podia. Existing podia are randomly inactivated (e.g. by capping protein binding [60]), i.e. their protrusion force is randomly switched off. As a consequence, they retract (actin depolymerization [58], [59]) due to their inherent contraction force which is assumed to be harmonic (i.e. proportional to podium length). In the end, sufficiently retracted podia are deleted. The number of podia is dynamically controlled by adaptation of the podium generation and inactivation probabilities (Modeling methods section B). The migration phenotype largely differs between cells with only one active podium (mostly ballistic movement with random turns) and cells with multiple active podia (mostly stretched out and resting with random reorientation moves).

The maximum speed of a podium (here, 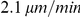; 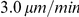 in [59]) is reached directly after podium generation. It is given by the ratio of protrusion force and podium-substrate friction. Similarly, the maximum speed of an entire cell is determined by the ratio of the total protrusion force (vector sum) and the total cell-substrate friction (podia plus cell body). The model thus exhibits podium traction dynamics as experimentally observed for cells on stiff substrates (‘frictional slippage’) [62]. The maximum podium length (here, 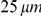; 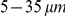 in [59]) is given by the ratio of protrusion force and podium contractility. It is reached in stretched out resting cells with two or more podia (Modeling methods section E).

Cell-cell alignment is achieved by first moving each podium independently according to its own dynamics and subsequently adjusting podia direction in order to avoid overlap between cells while preserving podium length. The podia adjustment used in this study is a basically pairwise approach. This can lead to conflicting requirements in dense cell culture with multiple neighbors resulting in partial cell-cell intersections. Cell-cell overlap is, however, also frequently observed in cell culture experiments.

**Figure 2:**
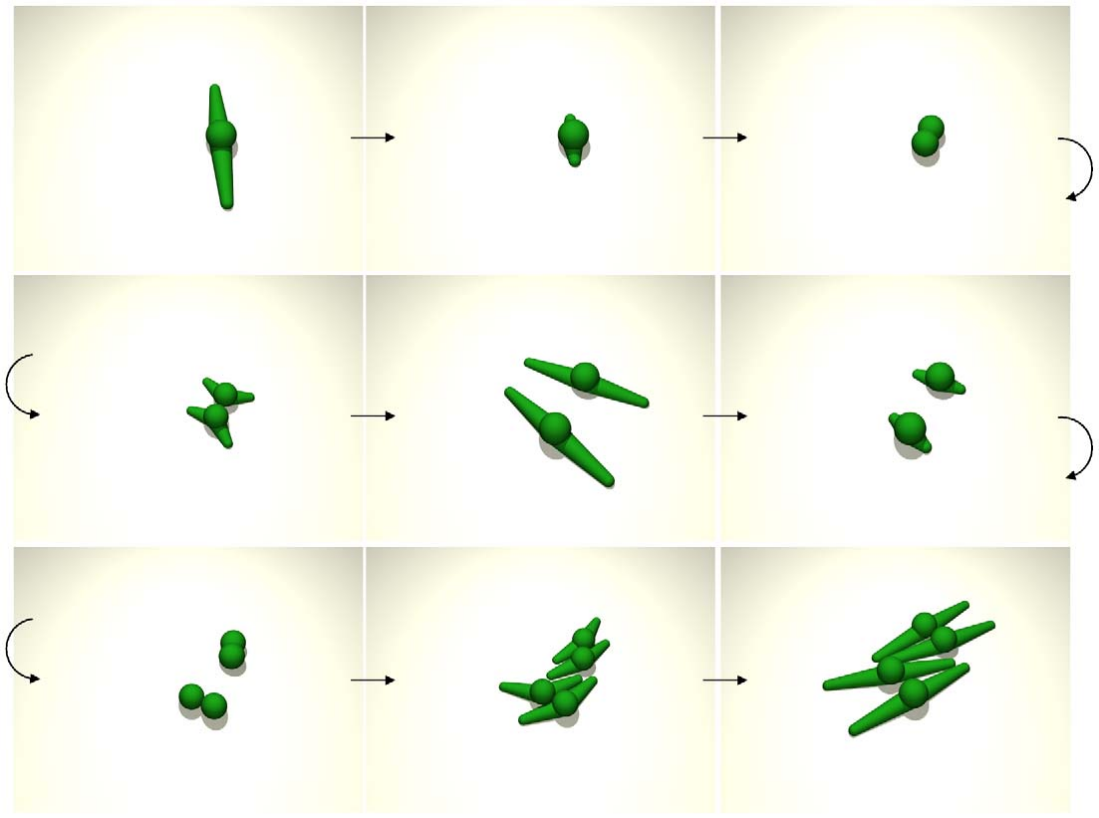
Cell division. Sequence of two cell divisions (top-left to bottom-right) for spindle-shaped cells with two podia. Podia are retracted prior to cell division and align to each other (see Modeling methods section C and F).

Podia of model cells retract prior to cell division and align to each other (Figure 2) as is also experimentally observed in proliferating MSC *in vitro* [63], [64] (and our own video material). Cell proliferation is modeled according to five cell cycle phases that involve four checkpoints for lower and upper cell volume, cell compression, and podia retraction, respectively (Modeling methods section F).

Further details of our podia model are given in Modeling methods sections B, C, and E. The model geometry is illustrated in Figure 15. A program flow chart is provided in Figure S7.

### MSC culture on plain substrates

**Figure 3:**
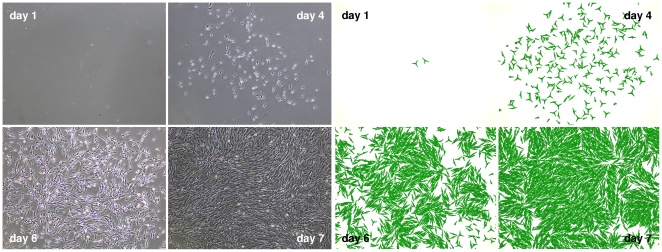
Cell culture. Images of an MSC population at day 1, 4, 6, and 7 of culture. Experimental (left) and simulation (right) results match qualitatively regarding cellular phenotype and quantitatively regarding spatial distribution and cell number (Figure 4). Model parameters, as also used for all other simulations of this study (if not stated otherwise), are given in Table 1.

In a first application our model is compared to our own experimental data on MSC expansion. Figure 3 displays images of MSC populations as obtained from experiment (left) and computer simulation (right) at day 1, 4, 6, and 7 of culture. Initially, cells disperse by fast migration and show fibroblastoid phenotypes with multiple podia. Subsequently, exponential population growth increasingly impedes cell migration and triggers cell-cell alignment. In this stage, cells exhibit a more spindle-shaped morphology. Finally, population growth ceases due to contact inhibition. The simulated cells mimic their biological equivalents by switching from a 3-podia fast moving phenotype showing frequent podia extensions and retractions at low cell density to a 2-podia phenotype with rare changes in podia line-up at high cell density. Analogously, cell proliferation is switched off in dense cell culture. This phenotypic and behavioral response is implemented by decreasing i) the number of podia, ii) the podia inactivation probability, and iii) the cell volume growth rate as the number of nearest neighbors increases. Details of our piecewise linear function approach are given in Modeling methods section G.

**Figure 4:**
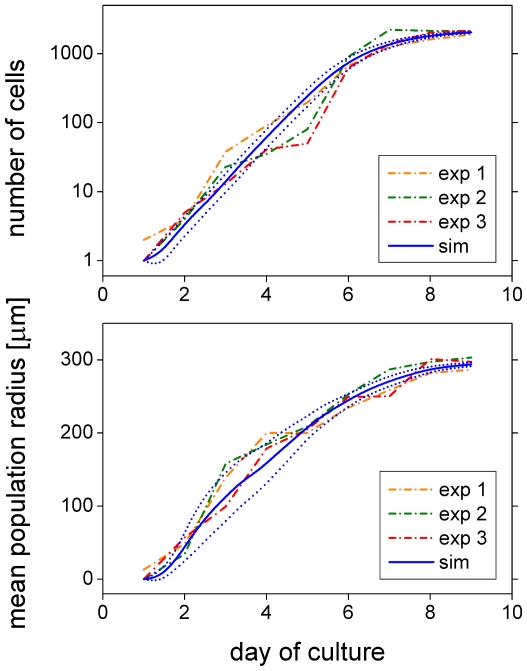
Cell culture growth dynamics. *In vitro* MSC growth in terms of the number of cells (top) and mean population radius (bottom) for three representative culturing experiments (dash-dotted) and the mean (solid) 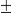 standard deviation (dotted) of 10 differently random-seeded simulations. Saturating exponential population growth and linear radius expansion are characteristics of MSC culture using Petri dishes. The mean population radius is confined to about 300 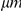 because of the experimental image dimensions. The experimental image frame is used for the evaluation of all simulation results of this subsection (MSC on plain substrates). Model cells are confined to a spherical area safely containing the image frame. This mimics the experimental effect of restricted expansion due to neighboring cell cultures (the effect depends on seeding density; an example is shown in Figure S1).

Figure 4 depicts the number of cells (top) and the mean population radius (bottom) as a function of cultivation time for the experimental (dashed) and the model (solid) system. The mean population radius is defined by 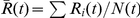 with 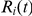 denoting the radial position of cell 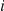 relative to the cell culture center and 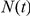 being the total number of cells at time 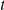. Experimental and simulation results are in good quantitative agreement. Even the higher variability of cell expansion early during cell culture is captured by the model (higher variability in mean population radius). A representative simulation run is shown in Videos S1 and S2 (close and far view, respectively). Model parameters as given in Table 1 were adjusted to fit the experimental data.

**Table 1:** Model parameter values.

description	parameter	value
cell-cell adhesion energy density	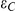	600 µN/m
cell-substrate adhesion energy/area	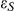	200 µN/m
Poisson ratio	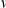	⅓
Young modulus	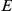	1 kPa
bulk compression modulus	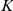	1 kPa
cell-cell friction constant	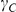	2.8 10^9^ Ns/m^3^
cell-substrate friction constant	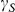	2.8 10^9^ Ns/m^3^
cell radius friction constant	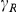	173 10^9^ Ns/m^3^
cell viscous friction constant	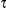	0.04 Ns/m
number of podia-related update rate	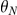	1440 1/d
independent podium update rate	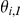	[2…0] 1/d
number of podia offset value	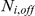	[3.5…2.5]
podium angle scaling constant	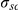	3.0 rad
protrusion force	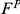	2.5 nN
podium-substrate friction constant	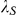	0.07 Ns/m
podium spring constant	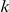	0.1 nN/µm
initial radius of cell body	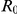	4.75 µm
target volume increment per time	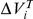	[1200…0] µm^3^/d
average number of volume increments per time	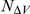	20 1/d
distance for nearest neighbor classification	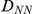	5 µm
number of nearest neighbors – proliferation	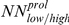	[4…5]
number of nearest neighbors – podia	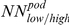	[1…3]

Numbers given in square brackets refer to values at low and high cell density, respectively, i.e. value at [low density … high density]. 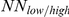 define the low and high cell density regimes, respectively (see subsection G). For parameter values related to the spherical cell bodies refer to our previous publications [34], [49], [50]. The number of podia-related update rate corresponds to the podia generation time of about one minute as measured for eukaryotic cells [53]. The maximum protrusion force of podia ranges between 0.5 and 10 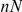 depending on cell type [94], [95].

**Figure 5 pone-0021960-g005:**
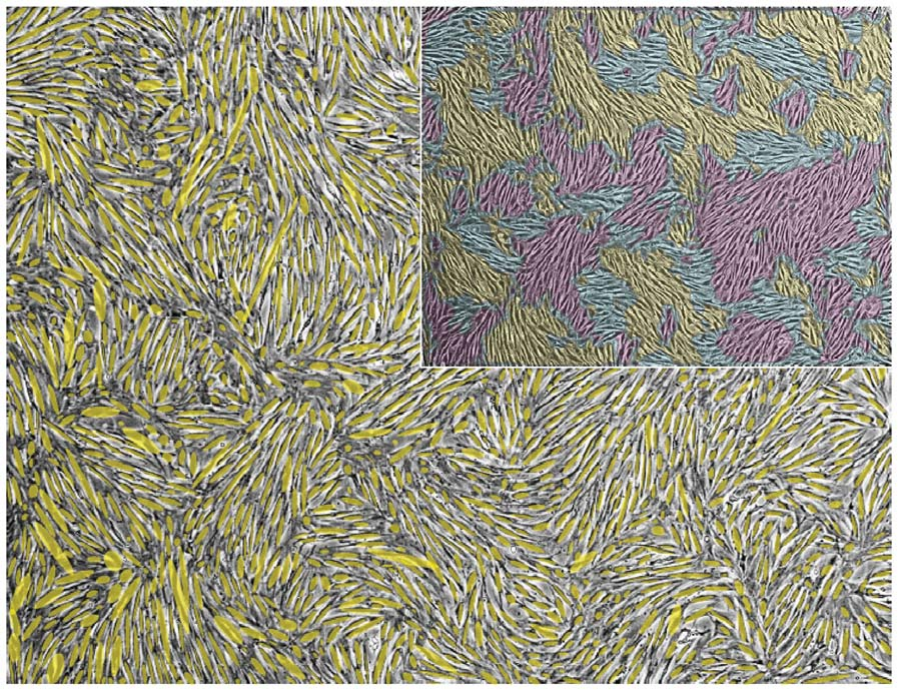
Cell orientation. Cell directional angles were determined by image segmentation and subsequent assignment of the segmented cell area principal axes. The figure shows the overlay of these principal axes as visualized by ellipsoids (yellow) and the corresponding experimental cell culture micrograph (gray). The inset shows patches of similar cell orientation for the same micrograph. They resemble magnetic spin (Weiss) domains.

The alignment of two cells can be quantified by the difference between their directional angles. Cell directional angles for the experimental data were obtained by image segmentation and subsequent determination of the main principal axes of the segmented cell areas. Coloring cells with similar orientation visualizes clusters of mutually aligned cells that resemble magnetic spin (Weiss) domains [65], [66], [67] (Figure 5). Interestingly, the existence of such clusters is also implied by the nematic theory of self-propelled particles [68], [69] stating that polar-ordered suspensions are unstable in low Reynolds number systems such as bacteria. Directional angles in the model are given by the consensus orientation of the podia as defined by the angle 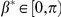 of the unit vector 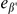 that maximizes the sum of the absolute values of the scalar products with the respective unit vectors 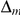 of the cell's 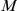 individual podia, i.e. 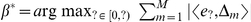.

**Figure 6 pone-0021960-g006:**
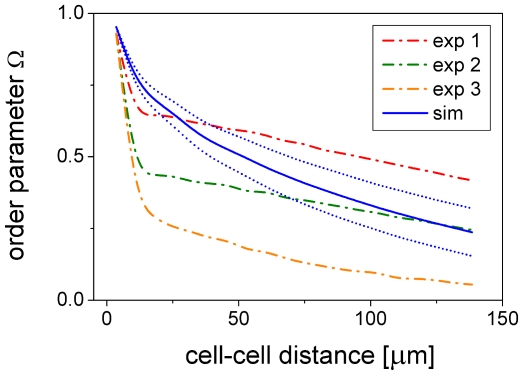
Cell-cell alignment. Order parameter 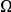 as a function of cell-cell distance at day 9 of cell culture for the same experimental data (dash-dotted) and simulations - mean (solid) 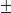 standard deviation (dotted) - as shown in Figure 4. Averaging was done over all pairs of cells within the picture frame.

Cell-cell alignment generally decreases with cell-cell distance. This is demonstrated in Figure 6 showing a decrease in the directional order parameter 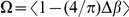 with cell-cell distance. Here, 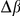 is the mean directional difference between two cells and 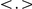 indicates averaging over all pairs of cells with a specific distance. 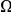 assumes the asymptotic values 1 for perfectly aligned and 0 for completely disordered cells. It is closely related to the order parameter 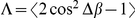 for isotropic-nematic transitions in two dimensions [70] that specifically relates to X-ray intensity measurements. In addition to 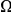, we define the order parameter 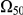 as the average of 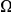 over cell-cell distances ranging between 0 and 50 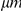. Obviously, our model slightly overestimates cell-cell alignment at small distances as compared to the image analysis results (Figure 6). This may be attributed to the fact that the experimental cell shapes generally deviate somewhat from ideal ellipsoids and that the shapes of model cells with two podia are much better defined. Nevertheless, there is a good overall qualitative agreement between model and experiment. In the following, we illustrate the influence of essential model parameters on model behavior.

#### Influence of cell proliferation

**Figure 7 pone-0021960-g007:**
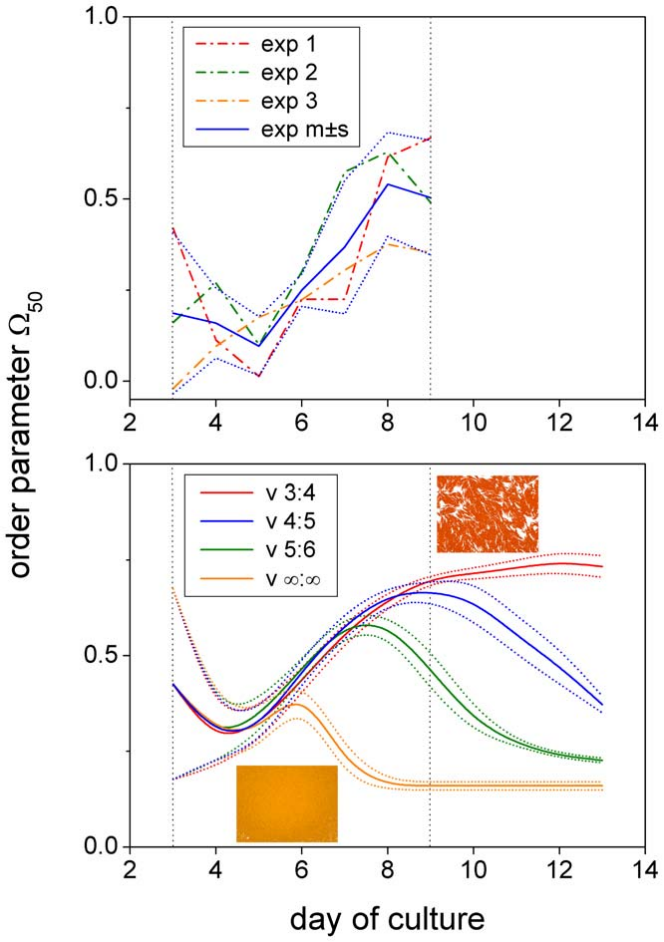
Density-insensitive cellular growth impairs cell-cell alignment. Order parameter 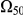 during culture days 3 to 13. Down-regulation of the mean cell volume growth rate from 1200 to 0 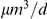 occurs linearly in the range between 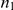 and 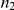 nearest neighbors (see Modeling methods section G) with 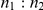 being set to 3∶4 (red), 4∶5 (blue), 5∶6 (green) and 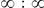
(orange) (i.e. down-regulation disabled). Mean values (solid) 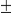 standard deviation (dotted) of 10 differently random-seeded simulations. Cell-cell alignment increases almost independently of cell growth during early cell expansion (>day 4) in the experiments (top) as well as the model (bottom). Subsequently, it may be destroyed again depending on how insensitive cell growth is with regard to cell density (i.e. the number of neighboring cells). The insets show simulation snap shots for regulation 3∶4 (red) and 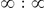
(orange) both at day 9 of cell culture.

*In silico* reproduction of our experimental results was most sensitive to model parameters that determine the down-regulation of cell proliferation and cell migration as a function of cell density (see Modeling methods section G). Delaying the down-regulation of cell growth to higher cell densities has little effect on the initial phase of cell expansion in which cell-cell alignment generally increases over time - experimentally as well as computationally (Figure 7). A similar increase in cell ordering was observed by Wu et al. [45] in myxobacterial swarming. In the course of cell culture, cell-cell alignment can either be maintained or be destroyed again depending on how efficiently cell growth is shut down by cell density. The destruction of cell-cell alignment occurs the earlier the more the down-regulation of cell growth is delayed to higher cell densities and the higher the absolute value of the volume growth rate. If down-regulation of cell growth is completely absent, a dense packing of cell bodies results after 7 days of culture (orange curve and inset in Figure 7, and simulation snapshot in Figure S2). An immediate decrease in directional order was only observed for very large volume growth rates (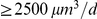) and with down-regulation of cell growth being completely disabled. We note that our simulations are best in agreement with experiment for down-regulation of cell growth at intermediate cell densities (blue curve in Figure 7). It is, however, not known whether the experimental curves would continue their final trend of slightly decreasing order (as also found in bacterial populations [67]) or settle at a fixed value. In case of settlement, our model could be adapted by assuming a progressive density-dependence. Nevertheless, we use the parameters of the best fitting model (Table 1; blue curves in Figure 4, 6, and 7) as the standard starting point for parameter variations. It is interesting to note that for this set of parameters the down-regulation of cell growth has no effect on spatial colony expansion implying that colony expansion is dominated by cell migration. Only when random podia activity is much reduced does cell growth show its accelerating influence on spatial colony expansion (Figure S3).

#### Influence of random podia activity

**Figure 8 pone-0021960-g008:**
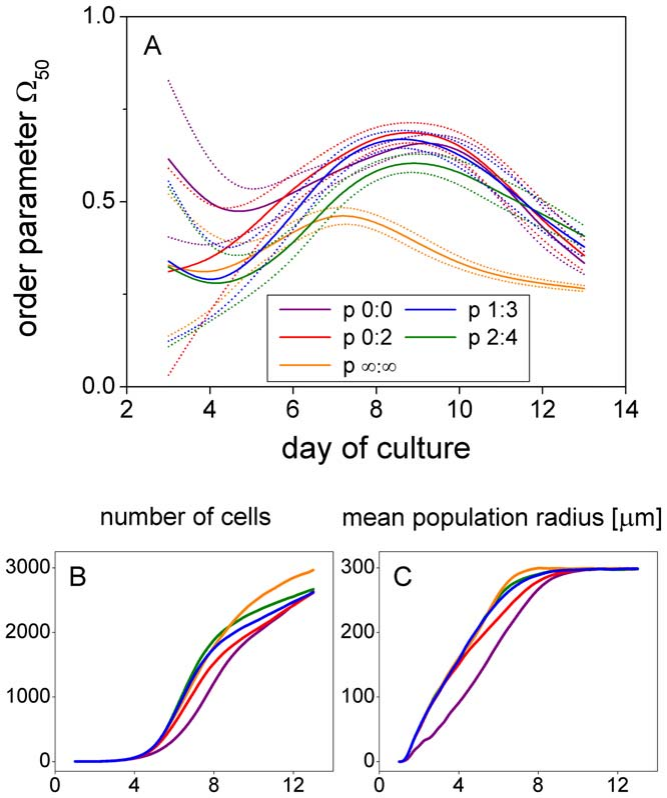
Density-dependence of cell migration is regulated to maximize cell-cell alignment and spatial colony expansion. Order parameter 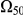 during cell culture days 3 to 13 (panel A). Down-regulation of cell migration occurs linearly in the range between 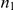 and 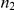 nearest neighbors (compare Figure 8). 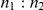 is varied from 0∶0 (violet; always 2 podia, no probabilistic podium inactivation, podium inactivation only due to cell division), over 0∶2 (red), 1∶3 (blue), and 2∶4 (green) to 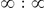 (orange; always 3 podia, probabilistic podium inactivation rate 2/d). The figure shows mean values (solid) 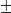 standard deviation (dotted) of 40 differently random-seeded simulations (the high variability of the results with respect to podia regulation during the first culture days required a higher number of samples as compared to all other figures). During days 6 to 11 cell-cell alignment (panel A) is best for regulations 0∶0, 0∶2, and 1∶3, whereas the mean population radius (panel C) is largest for 1∶3, 2∶4, and 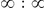. Thus, regulation 1∶3 is optimal in that it results in nearly maximal cell-cell alignment and spatial expansion at the same time. The number of cells (panel B) is not maximized because cells tend to spread out and acquire a certain contact area. The higher cell number obtained for 2∶4 and 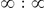 (>day 8) is paid for by higher disorder and necessarily less extended cells: the mean podium length (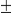
standard deviation across simulations) at day 9 of culture decreases from 23.9 (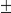
0.4), over 22.0 (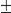
0.6), to 18.3 (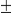
0.4)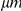 for regulations 1∶3, 2∶4, and 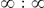, respectively.

Shifting the down-regulation of random podia activity to lower cell densities generally increases cell-cell alignment in our model. We define random podia activity by i) temporary changes in the number and position of podia due to probabilistic podium inactivation (independent podium inactivation rate, not being linked to podium inactivation prior to cell division) and ii) changes in the number of podia phenotype (number of podia offset value). Down-regulation of random podia activity thus means that the independent podium inactivation rate and the number of podia offset value are simultaneously decreased with cell density. Delaying the down-regulation of random podia activity to higher cell densities generally results in less aligned cells, faster spatial colony expansion, and higher cell numbers (Figure 8). Our results strongly suggest that the specific density-dependence of random podia activity found to fit the experimental data is optimal in that it results in a close to maximum cell-cell alignment and spatial colony expansion at the same time. The number of cells, however, is not maximized *per se* since cells tend to spread out and acquire a certain contact area with the substrate. The higher cell number obtained for more density-insensitive down-regulation of random podia activity is thus paid for by higher disorder and necessarily less extended cells as evidenced by a corresponding decrease in mean podium length (see caption of Figure 8).

#### Influence of cell-substrate friction

Faster spatial colony expansion is not essentially linked to faster cell population growth as could be presumed based on reduced contact inhibition. This is demonstrated by the variation in spatial colony expansion brought about by changes in cell-substrate friction. These had no effect on the number of cells and resulted in similar cell-cell alignment (Figure S5). As a consequence, the higher number of cells found for more density-insensitive down-regulation of random podium activity in the previous paragraph cannot be assigned to a reduction in contact inhibition but is indeed due to increased disorder and a concomitant reduction in cell extension.

#### Influence of podium length

**Figure 9 pone-0021960-g009:**
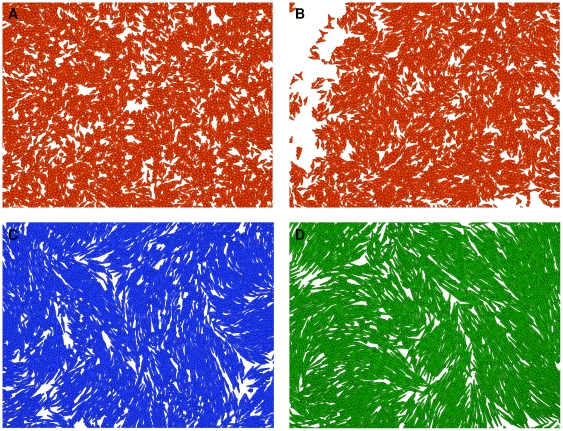
Homogeneity and size of alignment domains depend on podium length. Images of simulated cell cultures with short (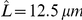, red, panels A and B), medium (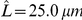, blue, panel C), and long (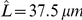, green, panel D) podia that form small, intermediate, and large alignment domains, respectively. Down-regulation of cell migration, i.e. simultaneous decrease in the number of podia from 3 to 2 (offset value from 3.5 to 2.5) and the independent probabilistic podium inactivation rate from 2 to 0/d, occurs linearly in the range between 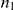 and 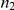 nearest neighbors. 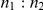 is set to 1∶3 for all simulations except for the one shown in panel B (short podia) in which it is set to 0∶2, i.e. down-regulation of migration already occurs at lower cell densities and results in better short range alignment (compare Figure 9). 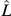 is the maximum length of a podium (Modeling methods section E).

**Figure 10:**
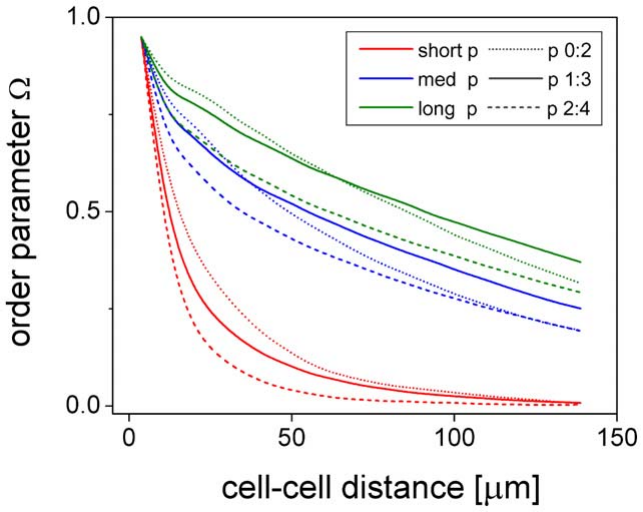
Cell-cell alignment increases with podium length. Order parameter 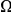 as a function of cell-cell distance for cells with short (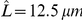, red), medium (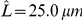, blue), and long (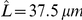, green) podia at day 9 of culture. The number of nearest neighbors for the down-regulation of cell migration (compare Figure 8) is set to 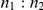 = 0∶2 (dotted), 1∶3 (solid), and 2∶4 (dashed). Cell-cell alignment is generally more accurate and of longer range for cells with longer podia. Moreover, short range alignment is better if down-regulation of cell migration already occurs at lower cell densities, i.e. lower values for 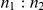. Note, however, that long range alignment can be less accurate in this case (medium and long podia). Shown are mean values of 10 differently random-seeded simulations.

Podium length has an effect on cell colonies in that cells with longer podia align more accurately and form larger alignment domains than cells with shorter podia (Figures 9 and 10). The impact of changes in podium length on cell-cell alignment generally diminishes with podium length. These findings are all in line with observations of Cho et al. [71] on self-organization in high-density bacterial colonies. Apart from cell-cell alignment, podium length also has an effect on colony expansion in that longer podia allow for faster expansion. Here, longer podia also result in a lower number of cells (lower cell density) since the image frame for evaluation is fixed and longer podia tend to occupy a larger substrate area (Figure S4).

In summary, our results indicate that the specific density-dependent down-regulation of cell growth and random podia activity found for the present data maximizes spatial colony expansion and cell-cell alignment at the same time. This strongly suggests that it is finally aimed at maximizing the number of cells per colony given the constraint that each cell seeks to maintain a defined area of contact with the substrate.

### MSC culture on micro-structured substrates

Next, we demonstrate our model's capability to reproduce experimental data on cell colony growth on micro-structured substrates. The alignment of cell podia to substrate microgrooves is modeled analogous to podia-podia alignment as used in the previous section (MSC on plain substrates). However, other than podia, grooves are never moved.

#### Parallel microgrooves

**Figure 11:**
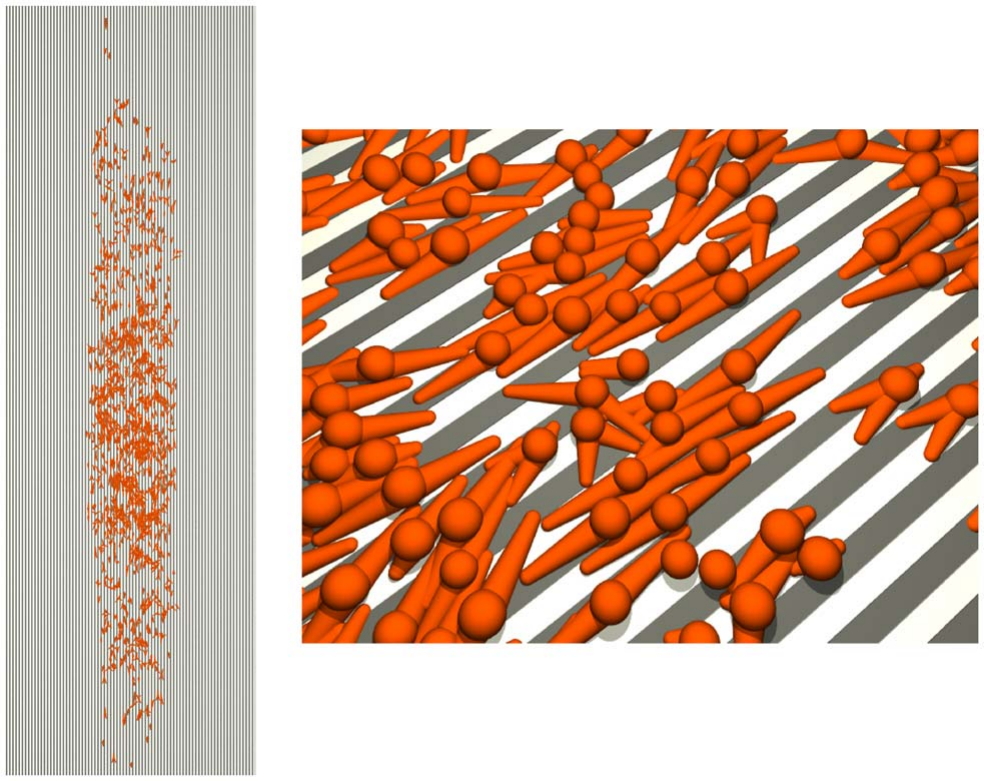
Growth on parallel microgrooves. Top view of a model cell population on day 4 of cultivation (left) and close up near its center (right). The pairwise alignment strategy applied for podia (see Modeling methods section C) does not result in perfectly aligned cells in highly crowded areas due to conflicting alignment requests (note that intersecting podia are also frequently observed in experimental cell culture). Microgrooves are modeled in 2D. Groove width and intergroove distance are both 8 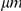. The podium-to-microgroove alignment probability per time step 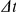 is 0.15 (see Figure 12 and Modeling methods section C). 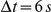 is used in all simulations of this study.

**Figure 12:**
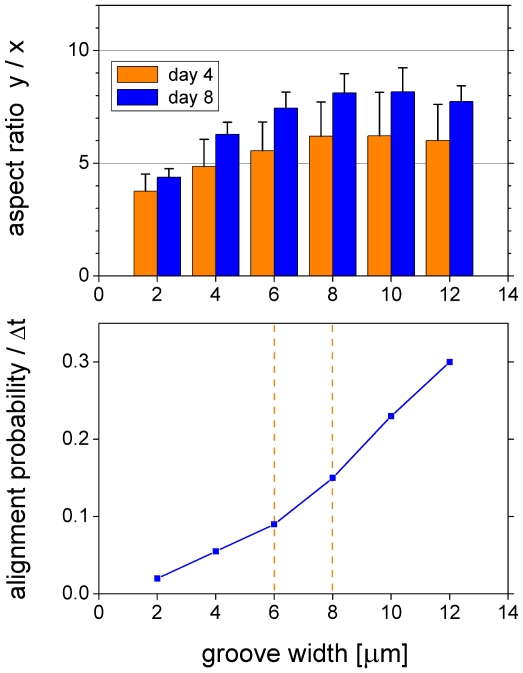
Colony aspect ratio on parallel microgrooves. Aspect ratio of the y-to-x mean colony extension for different groove widths at day 4 and 8 of cell culture (top) (mean and standard deviation of 10 differently random-seeded simulations). Our results match the experimental data of Ricci et al. [72] (their Figure 10) quantitatively. Podium-to-microgroove alignment probability per time step (bottom, 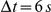) as used to reproduce the experimental results of Ricci et al. [72]. It compensates for the loss of alignment due to groove widening and must vanish as the groove width approaches zero (see text). The cross-over between the two different slopes occurs in the range where the grove width matches the width of the conically modeled podia (mean cone width 7 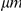; vertical orange lines).

An illustration of a parallel microgroove simulation is shown in Figure 11 and Videos S3 and S4 (close and far view, respectively). Figure 12 shows the aspect ratio of mean colony extension as a function of groove width. The aspect ratio is defined by 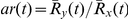, with 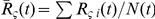 and 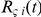 denoting the distance of cell 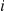 from the center of the cell population along the 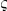-coordinate. Our results as presented in Figure 12 (top) are in good quantitative agreement with the data of Ricci et al. [72] on rat tendon fibroblast and bone marrow cell colony growth (their Figure 10). In order to reproduce their experimental results it was necessary for us to introduce a probability for the alignment of podia to microgrooves that increases with groove width (Figure 12, bottom, and Modeling methods section C). The increased cellular sensitivity for wider spaced microgrooves compensates for the loss of cell-to-microgroove alignment due to the same groove widening. We suggest that it is connected to the podia's ability to deform into the groove shape or sense unattached cell parts, just analogous to the fine feeling of fingertips. Accordingly, the alignment probability must vanish as the groove width approaches zero (plain substrate). Interestingly, the slope of the alignment probability changes where the groove width matches the width of the podia (Figure 12, bottom). Similar to Ricci et al. [72] (their Figure 11) although less pronounced we find a notably reduced colony growth on 8 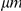-microgrooves (*micro*) between day 4 and 8 as compared to plain substrates (*plain*) and measured by colony area increase (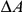) (*micro*: 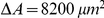, *plain*: 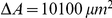). However, our simulations indicate that this reduction is mainly due to an initially faster and subsequently slower expansion with similar final outcome regarding colony area (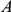) as well as cell number (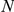) (*micro*: 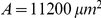, 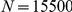, *plain*: 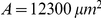, 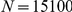). These results demonstrate the ability of our model to accurately account for cell alignment to microgrooves.

#### Starlike microgrooves

**Figure 13:**
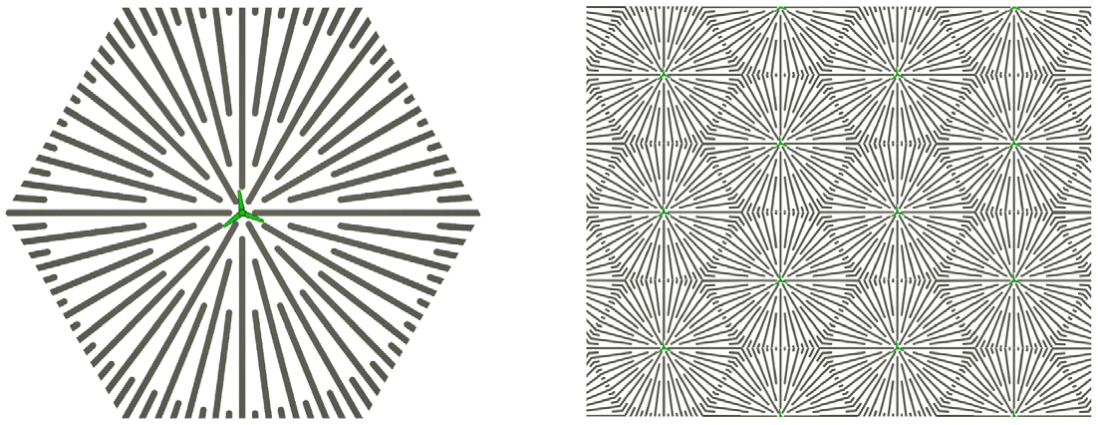
Starlike micropattern. Basic starlike plating unit (left) and schematic of the corresponding hexagonal lattice assembly (right).

**Figure 14:**
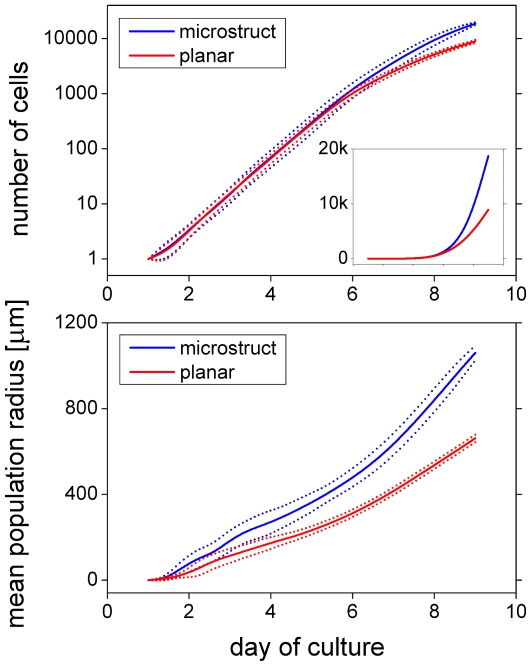
Growth dynamics on plain and starlike substrates. Characteristics of simulated *in vitro* MSC growth on a starlike micro-structured (blue) and plain (red) substrate in terms of the number of cells (top) and mean population radius (bottom). Mean 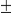 standard deviation of 10 differently random-seeded simulations. The cell yield after 8 days of culture is more than doubled for the micro-structured substrate (∼8900 versus ∼18700 cells). The results are calculated for a single large plating unit (geometry as in Figure 13, left; free growth and evaluation of all cells, i.e. no confinement to a spherical area and no picture frame for evaluation as used in Figures 4–10; see caption of Figure 4). The groove width and the intergroove distance are both 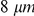 and the podium-to-microgroove alignment probability per time step is 0.15. The inset shows the number of cells on a linear scale.

Finally, we apply our model to predict a microgroove structure that increases the number of cells that can be harvested from cell cultures seeded by a single cell. The predicted structure is a hexagonal lattice of starlike micro-structured plating units as shown in Figure 13. The effect observed in our simulations was a more than two-fold increase in cell number after 8 days of cultivation (Figure 14). This effect is due to the microgrooves biasing the cells towards radial migration, thus avoiding circular movement that does not contribute to colony expansion. The increased spatial expansion results in later contact inhibition and hence higher cell yield (Figure 14). The dynamics are illustrated by Videos S5 and S6. The more directed colony outgrowth for the starlike micropatterned substrate can best be recognized by comparing Videos S2 and S6 (far view of plain and starlike micro-structured substrates, respectively).

## Discussion

On short time and length scales migrating cells are observed to move according to ballistic as opposed to diffusive dynamics. The cross-over between ballistic and diffusive dynamics occurs at time scales of 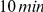 and length scales of 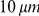 [40], [73], [74]. This behavioral difference has been suggested to be important in tissue formation, e.g. during vascularization or wound healing [30], [75]. It is built into our model on a first principle basis and shows the same general behavior and cross-over regime (Figure S6). Nevertheless, our model shows somewhat more ballistic and accelerated dynamics as compared to the experimental results of Dieterich et al. [40]. This can, however, largely be attributed to differences in cell-type and culturing conditions, or may results from the data fitting methods applied [41]. Apart from a better representation of single cell movement, our model is able to display directional cell-cell alignment of MSC in culture. This is prerequisite to accurately accounting for functional cell-cell contacts that form during colony growth and tissue formation and essentially feed back to cell development and cell migration [26], [27].

Computational models of cellular self-organization improve our understanding of tissue formation and will contribute to the development of optimal cell-based therapies in regenerative medicine. Here, we focused on MSC expansion *in vitro*. By monitoring and simulating MSC expansion it became evident that dynamic cell growth, cell migration, and cell shape transformations strongly impact the spatio-temporal organization of MSC. A related recently published study on the spatial heterogeneity of primary human myoblasts *in vitro* provides only qualitative predictions of cell behavior [76]. Thus, to the best of our knowledge, a detailed and quantitative description of the abovementioned cell processes are not considered by any other lattice free large scale IBM up to now.

Notably, a main feature of our model, the regulation of the podium inactivation probability (here as a function of cell density), has been shown be the basic mechanism of cell directional motion in chemotaxis [53]. Apart from this general consistency between model and experiment, probabilistic podium inactivation appears to regulate the noise that underlies cell movement. Consistent with this interpretation, cells are attracted to low noise states, i.e. states in which their podia are stretched out more permanently: cells move away from randomly inactivated towards steadily activated podia. These cell motion-related findings support our previous hypothesis of noise regulation being an important feature in cellular search and decision processes [77]. They suggest that the concept of noise regulation is not only applicable to molecular level processes like transcription and translation but scales up to the cell movement level. Noise regulation may also be applicable to basic organismal movement like foraging, invasion, and mating within a common framework of movement science [48], [78], [79]. Specifically, the step size of the random Lévy flights typically used to describe individual movement might depend on population density and position within heterogeneous environments.

Another basic question raised by our study is how cells sense properties of substrate micro-structures (here, width and direction of microgrooves) on a molecular level and adapt their behavior accordingly. Ensemble physical properties of our model, like cell orientational phase transitions, collective motion, and giant number fluctuations, as found in nematic systems of self-propelled particles [48], [65], [66], [68], [69], [80], would make a further tantalizing research direction.

The ‘tool box’ of cell migratory behavior newly introduced in this study substantially enhances the bandwidth of IBM in computational biology. The high sensitivity of our simulation results on model parameters regulating the density dependence of cell proliferation, migration, and morphology emphasizes the importance of cell-cell interactions in tissue formation. While density dependence of colony formation is a well known characteristic of MSC culture, studies on the impact of cell-cell contacts on MSC expansion are presently still rare. This may be related to the difficulties of non-invasive long-term single cell tracking in MSC culture, which is largely being disabled by cell clustering (JPK, unpublished results). In contrast, effects of substrate stiffness and micro/nano-structure on the growth, migration, and differentiation of MSC populations are studied in an increasing number of experimental studies [2], [18], [19], [20], [21], [28], [72], [81]. The microstructure we proposed for enhanced cell harvest will likely require advanced lab technology (such as robotics, microfluidics, or landscaped arrays) to initially place the seeding cells at the centers of the starlike plating units in production-scale culture. However, the advantage of a shortened *in vitro* cultivation phase, and thus a reduced probability of cell transformation [82], [83], will fairly balance this effort in therapeutic application.

In summary, we developed an improved model of proliferating and migrating fibroblast-like cell populations that explicitly represents cell podia and accounts for adjustment of cell behavior in response to cell density. We demonstrated that this model can quantitatively reproduce the spatiotemporal organization of MSC *in vitro* on plain as well as micro-structured substrates and proposed a microstructure that according to our simulations can significantly enlarge cell harvest in biotechnological applications. We think that our model has high potential for further development and can be evolved to accommodate individual cell behavior and collective dynamics of a variety of cell types and tissues in computational systems biology.

## Methods

The methods section consists of three mayor parts: Experimental materials and methods, Image analysis methods, and Modeling methods.

### Experimental materials and methods

Isolation of primary MSCs from sheep and cultivation of the cells: Bone marrow aspirates of 20 ml were obtained from the iliac crests of Merino sheep as previously described in [84]. The animals were treated in accordance with applicable animal protection laws (§ 8 Section 1). Authorization by the local legal representative (Regional Administrative Authority Leipzig, Germany) was granted: permit number 24-9168.11-Nr. TVV 18/06. Specifically, mononuclear cells were isolated from the heparinized aspirates (500 I.E. per ml; Ratiopharm Ulm, Germany) by Ficoll density gradient centrifugation (density 1.077 g/ml; Biochrom, Berlin, Germany) and plated at 2×10^4^ cells/cm^2^ in tissue culture flasks with Dulbecco's modified Eagle's medium (DMEM; Gibco, Karlsruhe, Germany) supplemented with 10% FCS, 100 U/ml penicillin, and 100 µg/ml streptomycin (both Biochrom). Cultures were maintained at 37°C in a humidified atmosphere containing 95% air and 20% O_2_–5% CO_2_ (Thermo Fisher Scientific, Dreieich, Germany). After 48 h, the non-adherent debris were removed by washing twice with phosphate buffered saline (PBS, Gibco, Karlsruhe, Germany) and incubated with fresh medium. Single clones of MSC were randomly chosen and used for daily phase-contrast microscopy (Olympus IX51, Hamburg, Germany) over a period of nine days.

### Image analysis methods

Experimental cell culture micrographs were segmented using fuzzy c-means segmentation [85]. The central point and main principal axis of each segmented cell region were used as cell center and cell direction, respectively. Further data analysis was conducted along the same lines as earlier described for the simulation results (see Results section).

### Modeling methods

The modeling methods section is subdivided into eight parts that explain the interaction between cell bodies as adopted from previous work [34], [49], [50], the introduction of cell podia, the 5-phase cell proliferation model, and the density dependence of cell proliferation, migration, and morphology. The specific subsections are: A) Interaction of cell bodies, B) Cell podia life cycle, C) Cell podia and their interactions, D) Integration of forces, E) Single cell properties of the model, F) Cell proliferation, G) Density dependence, and H) Table of model parameter values. Visualization of modeling results was obtained by using the freely available software packages POV-Ray 3.6 and ImageMagick 6.6.6-5. The basic source code is obtainable from the authors on request.

#### A) Interaction of cell bodies

The newly introduced cell model builds on a previous individual cell-based model of computational tissues as introduced by Galle and Drasdo et al. [34], [49], [50]. In this model cell bodies are assumed to interact with each other and the substrate according to adhesion and mechanical forces between elastic objects. The basics of this model are described in the following.

**Figure 15:**
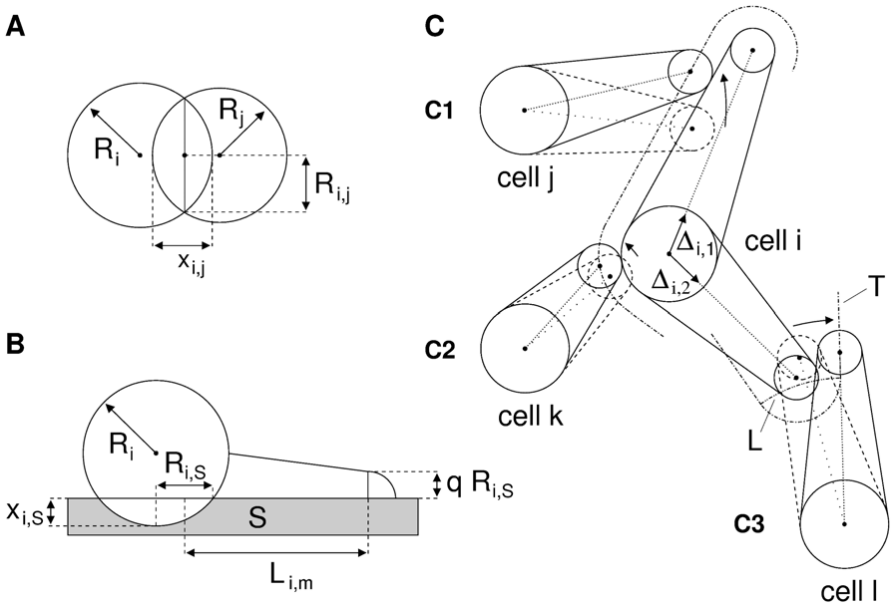
Model geometry. **A:** Radii 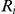 and 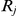 of cells i and j, respectively, the sum of their spherical cap heights 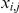 and the radius 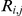 of their contact area 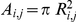. **B:** Side view of cell i on substrate 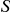 with full cell radius 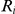, substrate adhesion radius 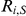, and substrate cap height 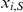. Podium cone geometry with base radius 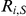, podium tip radius 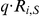 (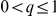) and length 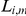 of podium m. The length is measured from half 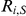 to the center of the podium tip. **C:** podium position updates (contact guidance) in 2D. C1: shift of podium 1 of cell j away from podium 1 of cell i. C2: shift of podium 1 of cell k away from the body of cell i for small overlaps (analogously, for the spherical podium tip). C3: move of podium 1 of cell l towards the tangent line (T) of podium 2 of cell i if the overlapping podium exceeds the length of the tangent section (center of cell l to tangent point). The limiting line (L) between cases C2 and C3 is a spherical section. For small simulation step sizes and low cell densities C1 and C2 will generally suffice. Otherwise case C3 is employed to move a podium off another cell. However, full removal requires subsequent application of C1 or C2. 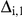 and 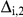 indicate the directional unit vector of podium 1 and 2 of cell i, respectively. The moved podia (cells j, k, l) are represented as their mid-line (dotted). Overlap between cells is defined by the intersection of this mid-line with the full podium of the respective resting cell (cell i) extended by the radius of the moved podium tip.

Isolated cell bodies are represented by elastic spheres of radius 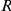. They flatten (deform) due to cell-cell and cell-substrate contacts that result from adhesion or mechanical forces (Figure 15). Cell deformation goes along with cell volume changes since cell bodies are assumed to be compressible (cell membrane permeability). The corresponding adhesion (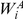), deformation (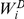) and compression (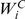) energies add up to the total body energy of cell 


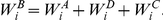
This energy depends on the distances between cells, the distances between cells and the substrate, and the cell radii. As a consequence, cell deformations in cellular aggregates equilibrate by either cell displacement or changes in cell radius. The individual energy terms for cell 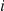 are given as follows. The adhesive cell-cell and cell-substrate energy reads
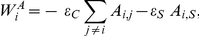
in which 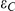 and 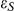 denote the adhesion energy per unit contact area between cells and between cells and the substrate, respectively. The actual contact area for cells 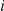 and 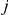 and cell 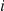 and the substrate 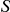 are termed 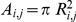 and 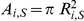, respectively (Figure 15). The deformation energy is approximated by the Hertz model
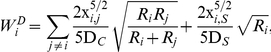
with 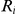 and 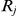 denoting the cell radii and 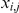
(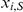) being the distance between cell 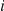 and cell 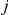 (cell 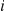 and substrate 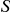) as explained in Figure 15. 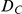 and 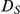 are defined through the Young modulus 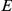 and the Poisson ratio 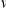 which are assumed to be the same for all cells
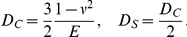
Finally, the compression energy is approximated harmonically as
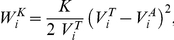
in which 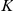 is the bulk compression modulus and 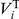 and 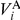 denote the actual and target volume, respectively (see subsection F). 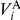 is the volume of the sphere with radius 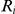 reduced by the sum of all spherical caps that overlap either with the neighboring cells or the substrate (Figure 15).

The body forces acting on the position of cell center 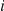 (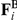) and the radius of cell 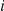 (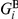) are calculated from the total body energy 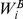 according to
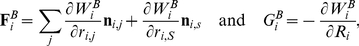
(1)respectively, with 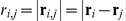 and 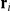 being the position vector of cell 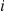. In the same way, 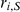 is the distance between cell 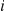 and the substrate 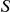. 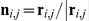 denotes the respective normal vector.

In addition to the above body forces, the model accounts for cell-cell and cell-substrate friction as well as friction associated with cellular reorganization during changes in cell radius according to
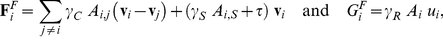
(2)in which 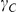 and 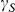 are the cell-cell and cell-substrate friction constants, 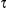 is the cell viscous friction constant with respect to the surrounding fluid, 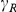 denotes the friction constant accounting for cell reorganization during radius changes, and 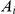 is the reduced cell surface area of cell 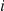 in which the spherical caps of cell-cell and cell-substrate contacts are replaced by the appropriate planar circular areas. The vectorial cell center and scalar radius velocities are denoted by 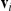 and 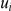, respectively.

The parameters specifying the physical interactions, i.e. the Young modulus 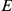, the Poisson ratio 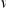 (alternatively the bulk modulus 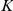), and the average adhesion energy per unit area 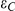 for cell-cell and 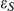 for cell-substrate contacts, are experimentally accessible, e.g. through different atomic force microscopy techniques [86], [87], [88]. The parameters specifying the friction forces can be estimated from e.g. cell sorting experiments [89].

#### B) Cell podia life cycle

The newly introduced cell model expands previous IBMs for computational tissues [34], [49], [50] by providing each cell with a variable number of podia that generate forces for cell movement. The life cycle of a podium starts with its generation and activation by a random process (i). Following its generation, the active podium elongates as a result of the protrusion force at the podium tip. This elongation builds up a traction force between podium tip and cell body (ii). The podium is inactivated by randomly switching off its protrusion force (iii). Subsequently, it retracts towards the cell body and is finally eliminated (iv). The individual steps are detailed in the following.

- *Random podium generation and activation:* Podia of cell 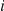 are generated according to the number of podia-related random update rate 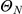 and the acceptance probability 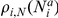 that depends on the actual number of active podia 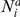. More specifically, if an update step is accepted according to 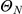 an additional podium is generated with probability
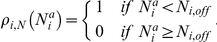
The generated podia have an initial length of half the adhesion radius 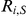 of the cell body (Figure 15). Podia angles are drawn according to a rejection probability 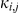 designed to avoid overlap with existing podia. First, the left and right nearest angular neighbors of the newly generated podium are determined and assigned a rejection probability 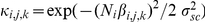, in which 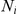 is the total number of podia (active and inactive), 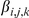 is the angle between the new podium 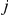 and its neighboring podium 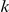 and 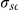 is a global scaling constant. The two probabilities are assumed to be independent and ‘or’-ed according to basic probability. The final rejection probability 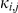 is obtained by rescaling to the full interval range 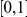. For cells with two and three podia as used in the present study the generated angles are consistent with the angular distribution of podium outgrowth peaking at 70° as published by Andrew and Insall [53].
- *Protrusion force of the podium tip and traction force between podium tip and cell body:* The tip of each podium is assigned a scalar protrusion force 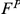 along the podium direction associated with actin polymerization and molecular motor machineries [58], [59]. A friction coefficient 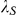 quantifies the friction between podium and substrate [62]. The traction force 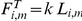 between podium tip and cell body is assumed to be harmonic with spring constant 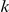. The podium length 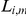 is measured from half the adhesion radius 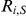 of the cell body up to the podium tip (Figure 15). The cell body is assigned a substrate friction coefficient 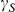 and an adhesion area 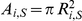. Assuming over-damped dynamics the equations of motion for podium 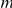 (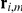) and the cell center (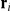) read
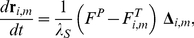
(3)and
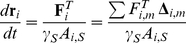
(4)respectively. 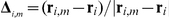 denotes the unit directional vector of podium 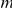.
- *Random podium inactivation:* Podia are inactivated according to the number of podia-related random update rate 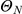 and the independent random update rate 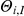. If an 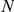-update occurs the 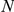-related inactivation probability is set to
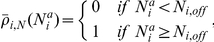
in which 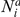 is the number of active podia of cell 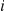. Otherwise 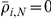. If an 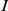-update occurs the 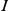-related inactivation probability is set to 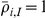. Otherwise 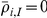. The inactivation probabilities are assumed to be independent and ‘or’-ed according to basic probability resulting in the final podium inactivation probability
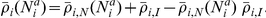
If inactivation is effected the podium protrusion force is permanently switched off. This may be related to e.g. binding of capping proteins to the actin filaments [60].
- *Podium retraction and final elimination:* With its protrusion force switched-off the podium retracts towards the cell body due to its intrinsic contraction force, mimicking actin depolymerization [58], [59]. Finally, it is eliminated when it hides below the cell body, i.e. when the podium length is smaller than the adhesion radius 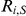. This is one reason for attaching the podium at half the adhesion radius. Another reason is that in round migrating cells podia are often not visible in a microscopic top-down view, i.e. podia are often indeed hidden below the spherical cell body.

#### C) Cell podia and their interactions

The present model simplifies cells by using a geometric ‘ball and stick’ representation and employing a basically pairwise cell-cell alignment approach. In regions of high cell density this can lead to temporary intersections between cells, which are, however, also frequently observed in cell culture.

Podia are represented as cones with spherical tips (Figure 15). Their main determinants are their cone tip and base positions as well as cone tip and base radii. The cone base position is set equal to the x-y-coordinates of the respective cell center. Accordingly, the cone base of cell 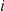 has a radius equal to the adhesion radius 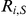. The cone tip has a radius of 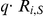 (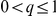). The interactions among podia and between podia and cell bodies are modeled as a two step process. First, all podia are independently updated according to their intrinsic forces. Second, the podia are shifted relative to one another to implement contact guidance. More specifically, the axis of each podium is checked for intersection with the podium and the cell body of its neighboring cells, and if so, moved off the cell with its length being preserved (Figure 15). The podia are not updated in a symmetric fashion, rather the movement activity is assigned to only one podium according to a global precedence scheme. Briefly, at the start all podia have precedence level one. In case of a podium-cone intersection the intersecting podium is moved if the intersection is small, i.e. the podium mid-lines do not intersect (Figure 15, C1). Otherwise the podium with the shorter distance between intersection point and its tip is moved. If a podium intersects a cell body the podium is moved (Figure 15, C2). If two podia hit head-on the shorter podium is moved (Figure 15, C3). The moved podium is assigned a precedence level one above that of the respective resting podium. This pairwise setting can have global impact since podia with a relatively higher precedence level are always moved. If a podium has multiple intersections with the same neighboring cell the ‘nearest’ move with the shortest distance between cell center and intersection point is applied. If a podium has multiple intersections with different neighboring cells this cell is temporarily ‘stalled’ (not updated). The precedence level of a podium is reset to unity if no intersection occurs. Switched off podia are never shifted.

Podium alignment to microgrooves is handled much like the podium-podium alignment described above with the microgrooves being ‘podia’ that are never moved. Another difference is that the alignment to microgrooves is not executed every time step but rather according to an alignment rate (probability per time). This enables the model cells to cross over microgrooves as is experimentally observed. This behavior is further facilitated by not executing alignment if the podium has already ‘climbed’ the microgroove, i.e. if its tip center is located within the microgroove.

#### D) Integration of forces

Podia dynamics are calculated according to equation (3) and the interaction rules described in the previous paragraph. The dynamics of the cell bodies are calculated according to the balance of forces (equations (1), (2), and (4))
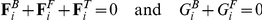



for the center and the radius of each cell 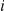, respectively. These equations are solved for the velocities 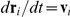 and integrated in time. Note, however, that they are coupled due to the fact that the friction forces 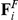 depend on the relative velocities of neighboring cells.

#### E) Single cell properties of the model

Here, we present the mathematical formalism for deriving the podia and whole cell movement properties used in the results section. The maximum elongation speed of a newly generated podium is 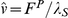 as calculated from equation (3). Similarly, the maximum podium length of a resting cell is 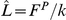. The podium elongation dynamics assuming an immobile cell body read 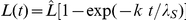. This is quantitatively similar to the one observed for the actin dynamics-based model of Mogilner and Rubinstein (their Figure 4B) [59]. The equilibrium speed and podium length of a cell with only one podium read
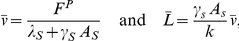
respectively (equations (3) and (4)). For a cell with two podia one being active and one being inactive (trailing) the equilibrium speed and podium lengths are
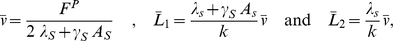
respectively. Hence, the equilibrium speed is reduced by the trailing podium as compared to a cell with only one active podium. Generally, the equilibrium speed is given by the ratio of total protrusion force (vector sum) and total friction. The length of a podium increases with cell velocity. If the length of a trailing podium is larger than the cell body adhesion radius it is not eliminated unless the active podium is inactivated or the cell slows down due to obstacles.

#### F) Cell proliferation

Cell proliferation is modeled according to five cell cycle phases. Simulations start with a cell volume 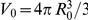 for each initializing cell. The following growth sequence is the same for cells directly after cell division. Hence, the description starts here. Directly after cell division daughter cells are assigned phase 0 and half the target volume 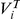 of their parent cell. They adjust their radius 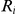 (and thus their actual volume 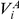) to match 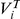 (lower volume checkpoint). Subsequently, cells enter phase 1 in which they proliferate according to an average growth rate that increments 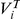 by 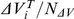 using an average number of volume increments per time 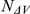 (probabilistic rate) (for general growth model properties see also [90], [91]). If the actual volume 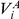 lags behind 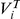, e.g. due to hindrance by neighboring cells, cells enter quiescence and are assigned phase 2. They reenter phase 1 if they relax their radius. If both target and actual volume have reached 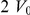 (upper volume checkpoint) cells enter phase 3 in which proliferation is stopped and podia are inactivated. This is motivated by experimental observations that dividing cells tend to decrease motility and break adhesive contacts with the substrate and neighboring cells [63], [64], [92]. If finally all podia are deleted (podia retraction check point) cells enter phase 4 and divide.

#### G) Density dependence

The density dependence of cell proliferation, migration, and the number of podia were most important for the present modeling results. Contact inhibition of proliferation and migration of fibroblast-like cells have been described and modeled by others before [92]. However, data on the density-dependent control of the number of podia are rare [93] and the process has not been integrated into individual cell-based models so far. In our model density dependent regulation is based on the number of neighboring cells 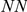 that are located within the vicinity of a given cell. This vicinity is defined by a preset distance 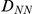 from either the cell's podia or cell body. A neighboring cell is within the vicinity of another cell when either one of its podia or its cell body is in the vicinity of the other cell. Cell proliferation (cell volume growth rate), cell migration (podium inactivation rate), as well as the number of podia (offset value) were defined as ramp functions, i.e. constant for low (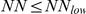) and high (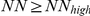) cell density, with a linear transition regime in between (see also Table 1).

#### H) Table of model parameter values

## Supporting Information

Figure S1 **Contact between two cell colonies at day 9 of culture.**
(TIF)

Figure S2 **Snapshot of a simulation at day 8 of culture with density-dependent contact inhibition of cell growth being disabled.**
(TIF)

Figure S3
**Cellular growth accelerates spatial colony expansion only if cell migration is low.** Mean population radius and number of cells (insets) during simulated cell cultivation. Down-regulation of cell migration as described in the caption of Figures 8 and 10 occurs either between 1 and 3 nearest neighbors (top) or is always down-regulated (bottom). Down-regulation of cell volume growth from 1200 to 0 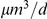 occurs linearly in the range between 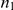 and 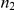 nearest neighbors, with 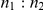 being assumed as 3∶4 (blue), 4∶5 (red), and 5∶6 (green). The results demonstrate that the growth rate impacts radial expansion only if cell migration activity is low. Thus, cell migration appears to generally dominate spatial colony expansion. Shown are mean values of 10 randomly seeded simulations.(TIF)

Figure S4
**Podium length accelerates spatial colony expansion and decreases cell number density.** Number of cells (top) and mean population radius (bottom) during simulated cell cultivation. The podium length is either short (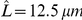, red), medium (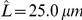, blue), or long (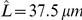, green). Cell growth rate and migration activity is down-regulated according to the standard set of parameters (Table 1). Larger podium length results in faster spatial colony expansion but a lower number of cells because larger podia occupy a greater substrate area and evaluation is performed for a fixed picture frame (cell density measurement). Shown are mean values of 10 randomly seeded simulations. The inset (top) shows the number of cells on a linear scale.(TIF)

Figure S5 **Cell-substrate friction accelerates spatial colony expansion but leaves the number of cells unaffected.** Mean population radius and number of cells (inset) for low (red), medium (blue), and high (green) friction corresponding to 0.5, 1.0, and 1.5 times the standard values used for cell body- and podium-substrate friction (Table 1). The observed faster expansion for lower friction has almost no effect on the number of cells. Shown are mean values of 10 randomly seeded simulations.
(TIF)

Figure S6
**Kinetic exponent for the mean squared displacement (msd) of single non-interacting cells over time for different probabilistic podium inactivation rates.** The kinetic exponent 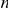 characterizes the evolution of the msd with respect to time 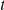, i.e. msd 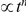. For pure diffusion 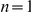, for movement with a constant velocity 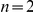, and for movement with a constant acceleration 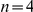. For model cells acceleration (force) is proportional to podium length which itself is a function of time. This can result in kinetic coefficients 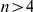 for individual cells. The figure shows mean values (solid) 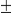
standard deviation (dotted) across 10 simulations running 12000 cells each. The curves vary with respect to the independent podium update rate 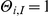 (red), 2 (blue), 4 (green), and 20/d (orange) that governs probabilistic podium inactivation and thus podium turnover. The curves generally show the same characteristics as the measurements of Dieterich et al. [40] (their Figure 1). Nevertheless, our results show higher values for the kinetic exponent and may thus overestimate ballistic cell movement and acceleration. In addition, the final increase of the kinetic exponent, only seen in the model cells for low and medium podium update rates, is an artifact of our model which is due to cells with trailing podia that move with constant velocity (Modeling methods section E). However, we checked that this effect is without consequences for the results of the main manuscript since cell-cell interactions (not accounted for in this figure) dramatically limit the cell travelling time and range.(TIF)

Figure S7 **Program flow chart.** In each time iteration step, first podia are moved, second bodies, and third radii. Finally, phenotype and proliferation states are updated. The underlying physical, heuristic, and random models are described in the methods section of the main manuscript. Each podium is elongated independently according to the physical model. Overlapping podia are aligned to each other according to the assigned pairwise heuristic. Podia are switched on or off according to random generation and inactivation rates, respectively, which depend on all podia of a given cell. Phenotype (number of podia offset value, independent podia inactivation rate) and proliferation (cell cycle phase, volume growth rate) states are regulated according to the local cell density (accounting for cell bodies and podia).
(TIF)

Video S1 **Plain substrate - close view.**
(MPEG)

Video S2 **Plain substrate - far view.**
(MPEG)

Video S3 **Parallel microgrooved substrate - close view.**
(MPEG)

Video S4 **Parallel microgrooved substrate - far view.**
(MPEG)

Video S5 **Starlike microstructured substrate - close view.**
(MPEG)

Video S6 **Starlike microstructured substrate - far view.**
(MPEG)
