# Supplementary figures and images for: Spatial Organization of Mesenchymal Stem Cells In Vitro—Results from a New Individual Cell-Based Model with Podia
Source: PLoS One. 2011 Jul 8;6(7):e21960. doi: 10.1371/journal.pone.0021960 (PMC3132757; doi:10.1371/journal.pone.0021960)

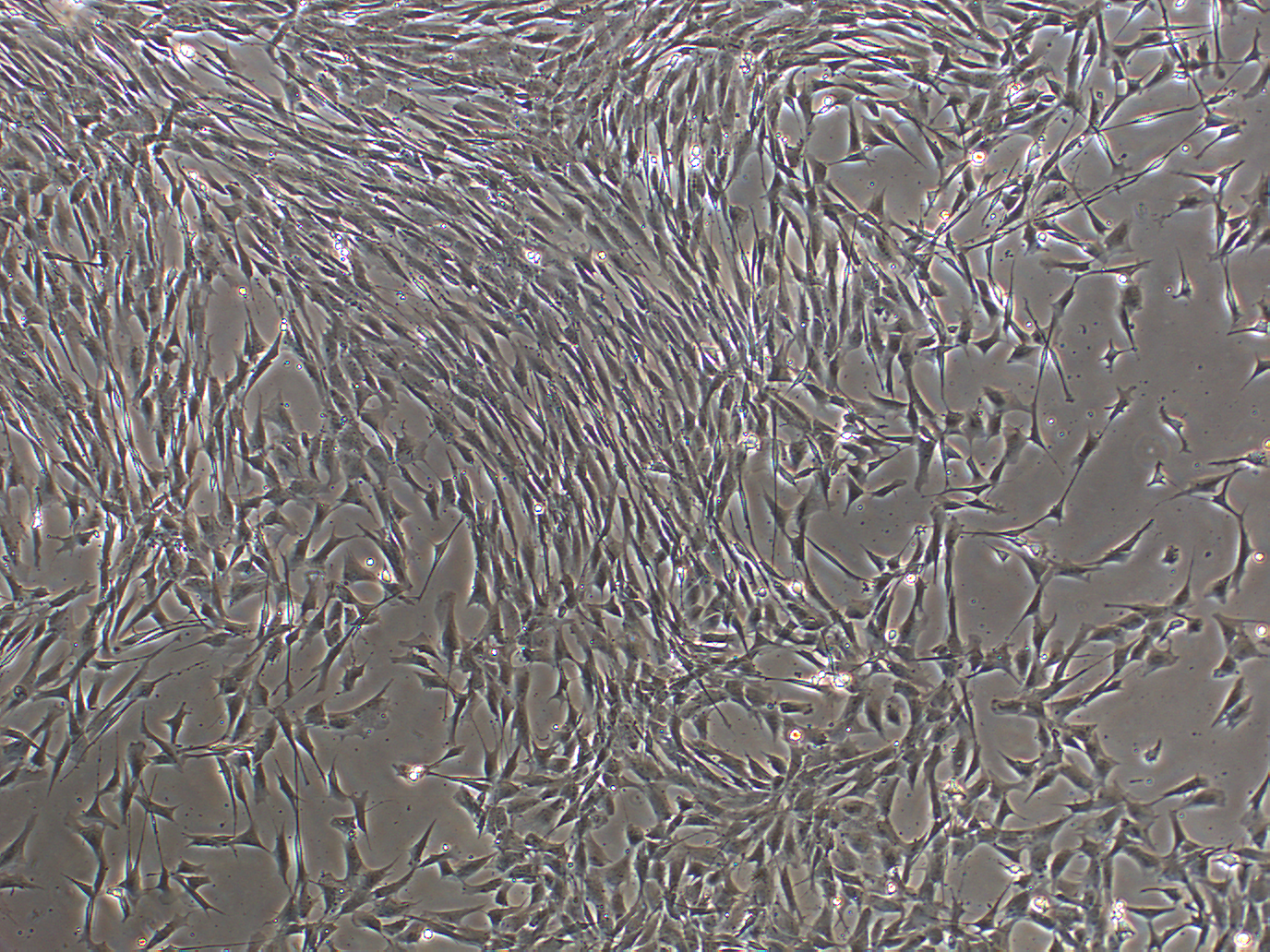

Supplement: Figure S1 — Contact between two cell colonies at day 9 of culture. (TIF) [file pone.0021960.s001.tif]

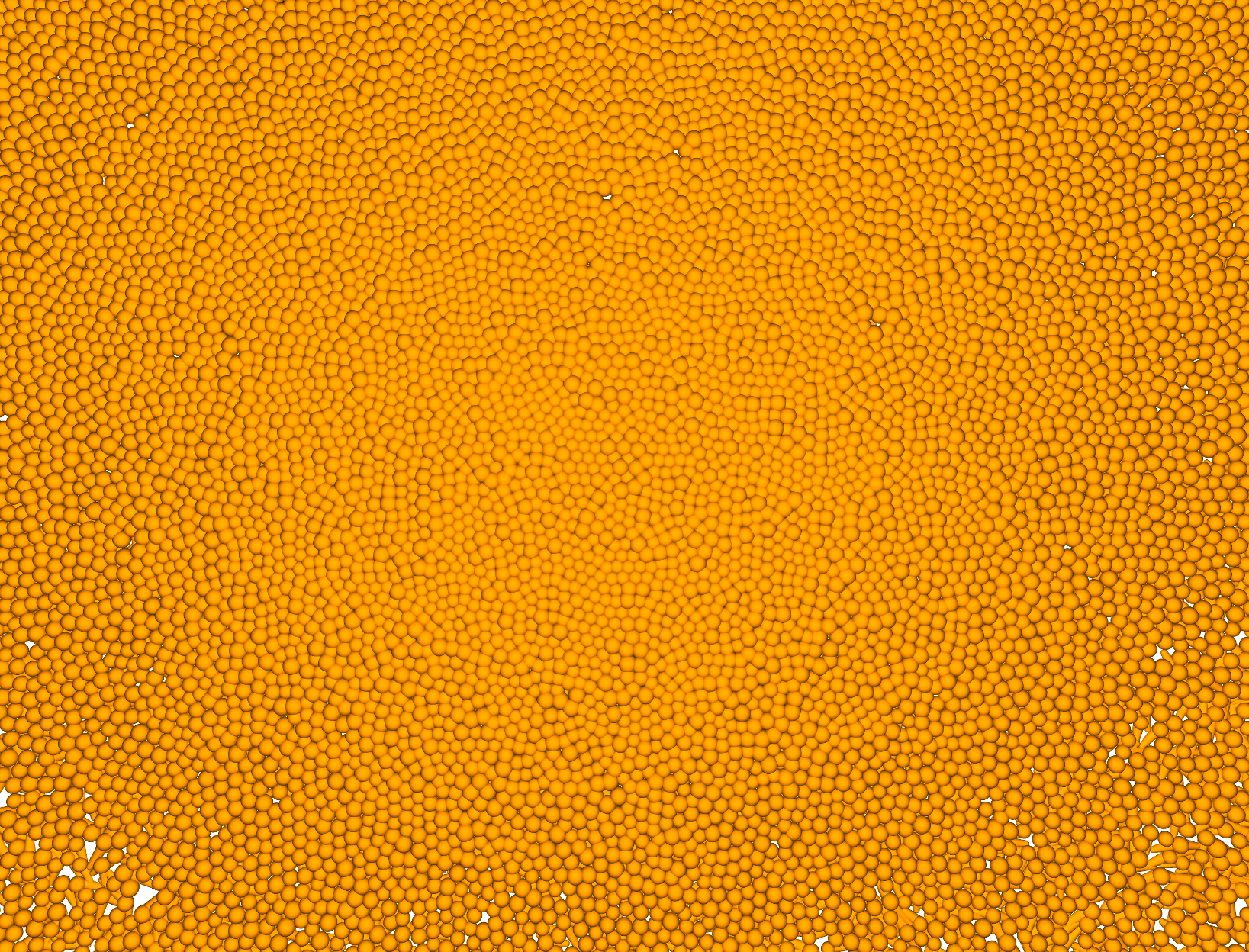

Supplement: Figure S2 — Snapshot of a simulation at day 8 of culture with density-dependent contact inhibition of cell growth being disabled. (TIF) [file pone.0021960.s002.tif]

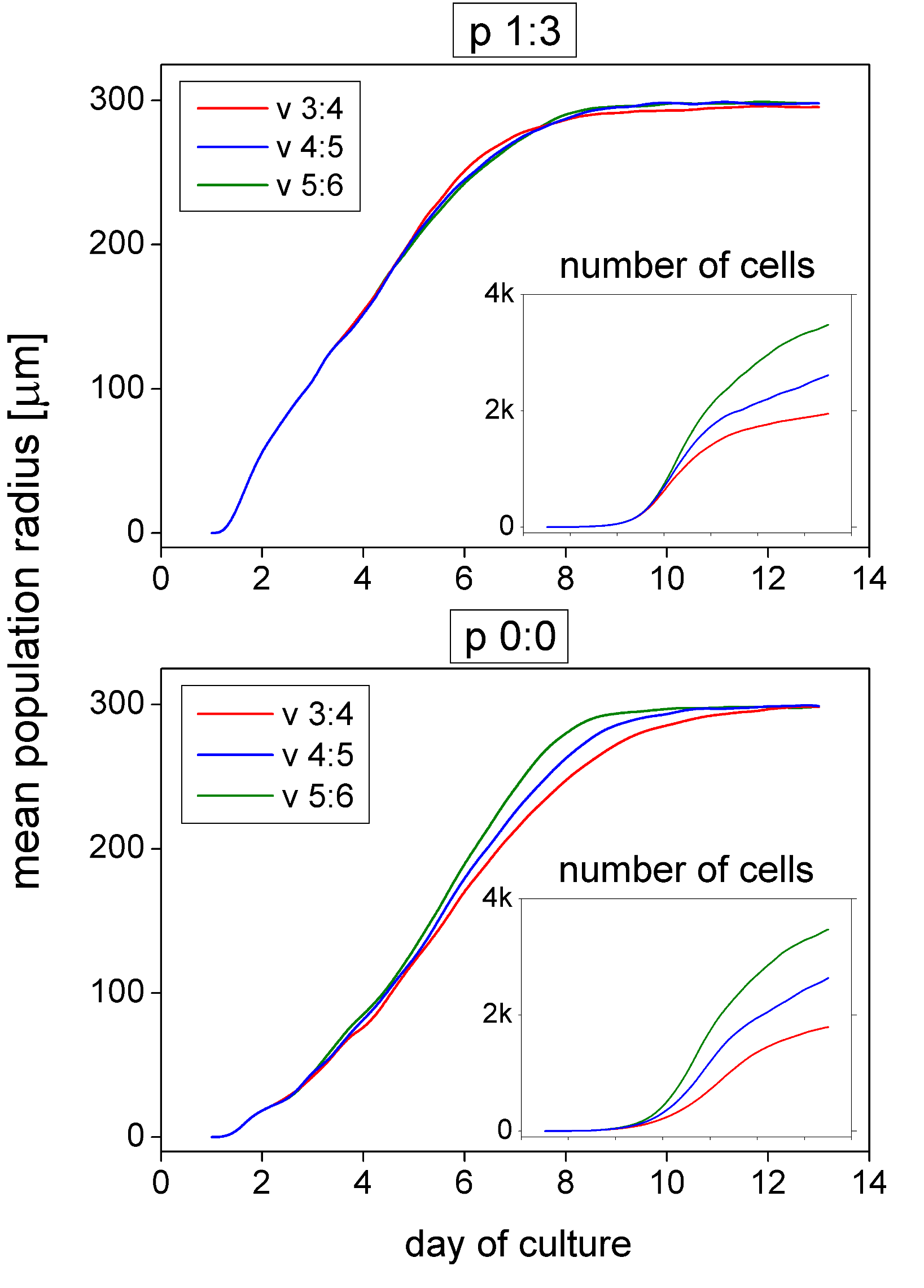

Supplement: Figure S3 — Cellular growth accelerates spatial colony expansion only if cell migration is low. Mean population radius and number of cells (insets) during simulated cell cultivation. Down-regulation of cell migration as described in the caption of Figures 8 and 10 occurs either between 1 and 3 nearest neighbors (top) or is always down-regulated (bottom). Down-regulation of cell volume growth from 1200 to 0 occurs linearly in the range between and nearest neighbors, with being assumed as 3∶4 (blue), 4∶5 (red), and 5∶6 (green). The results demonstrate that the growth rate impacts radial expansion only if cell migration activity is low. Thus, cell migration appears to generally dominate spatial colony expansion. Shown are mean values of 10 randomly seeded simulations. (TIF) [file pone.0021960.s003.tif]

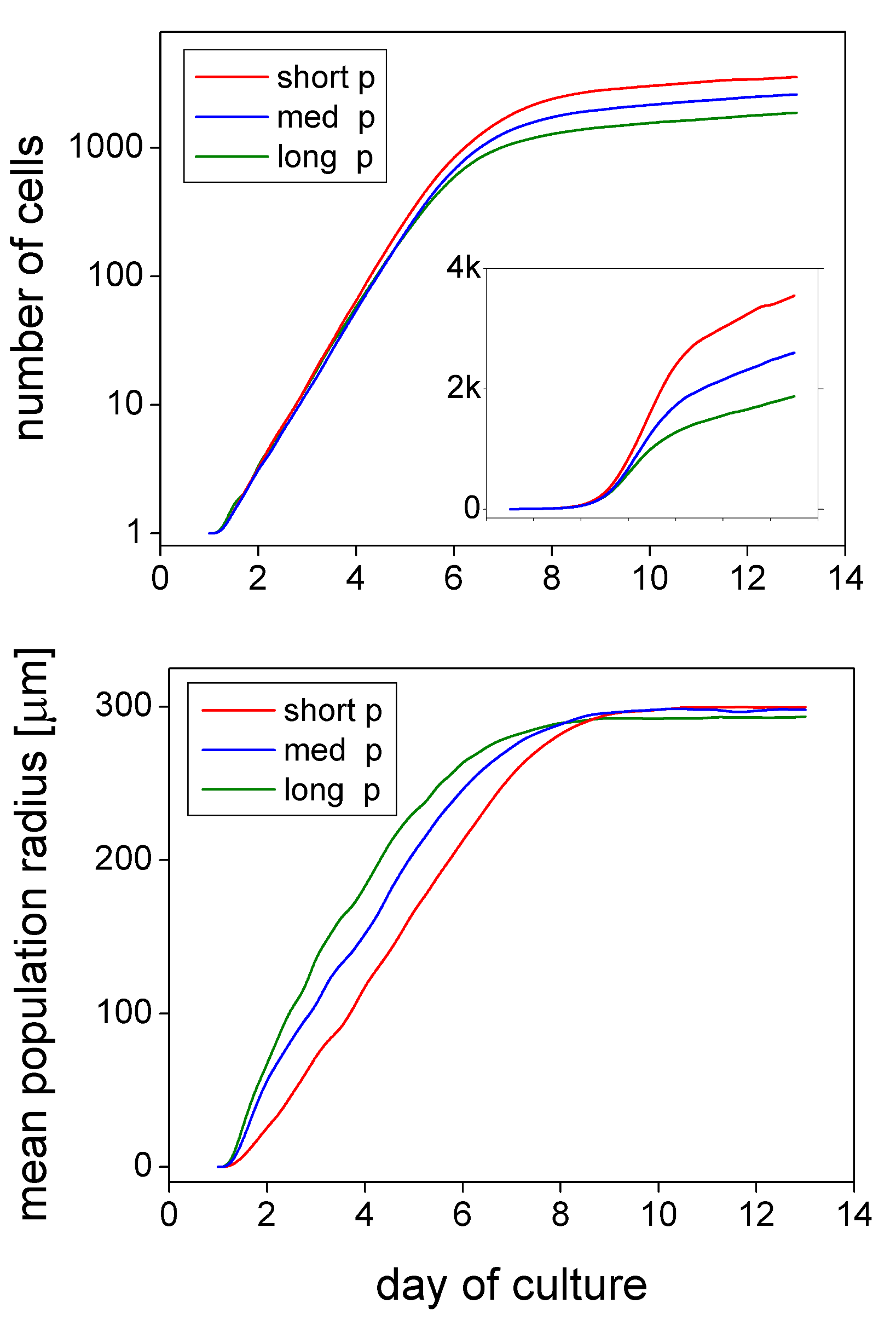

Supplement: Figure S4 — Podium length accelerates spatial colony expansion and decreases cell number density. Number of cells (top) and mean population radius (bottom) during simulated cell cultivation. The podium length is either short (, red), medium (, blue), or long (, green). Cell growth rate and migration activity is down-regulated according to the standard set of parameters (Table 1). Larger podium length results in faster spatial colony expansion but a lower number of cells because larger podia occupy a greater substrate area and evaluation is performed for a fixed picture frame (cell density measurement). Shown are mean values of 10 randomly seeded simulations. The inset (top) shows the number of cells on a linear scale. (TIF) [file pone.0021960.s004.tif]

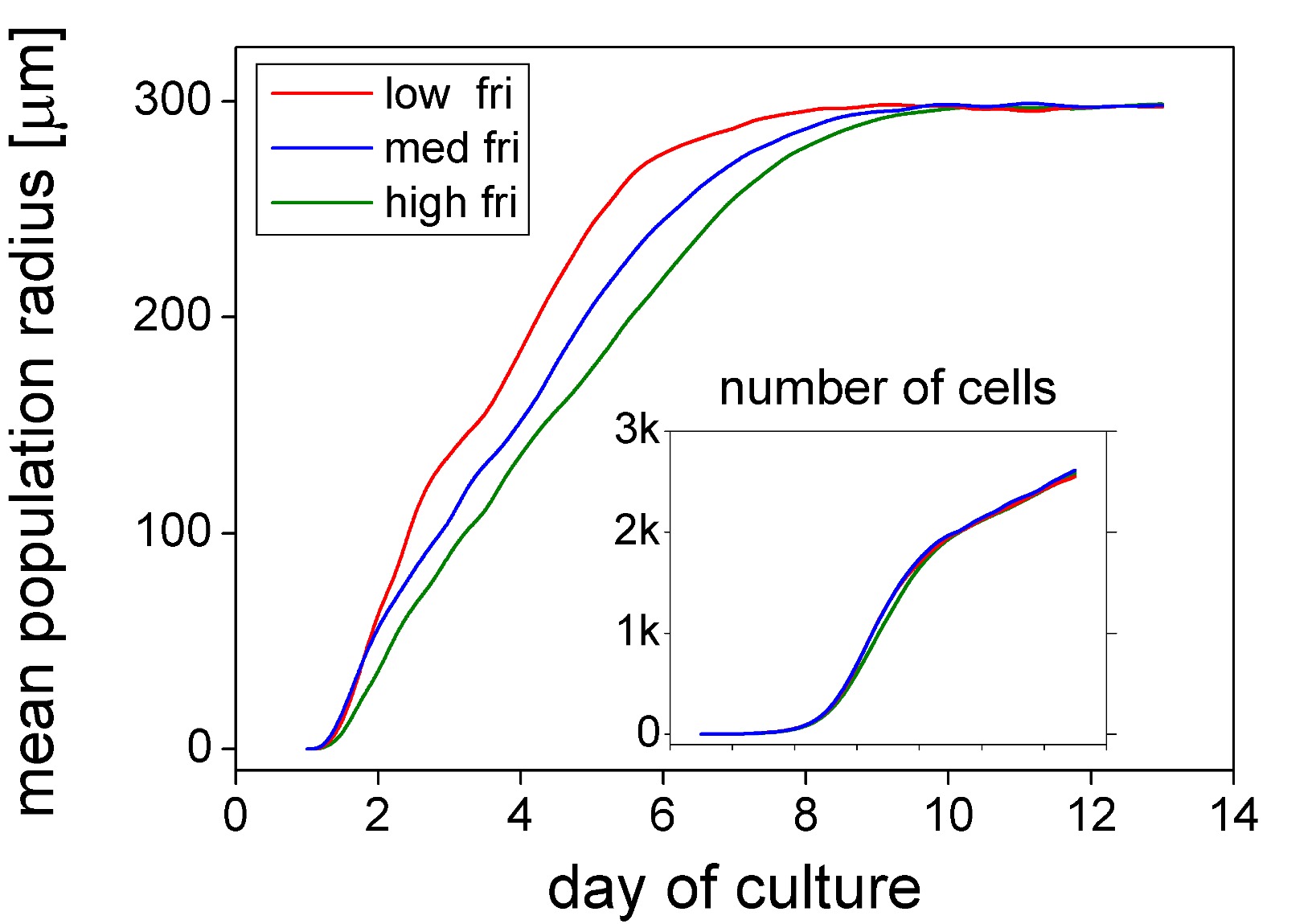

Supplement: Figure S5 — Cell-substrate friction accelerates spatial colony expansion but leaves the number of cells unaffected. Mean population radius and number of cells (inset) for low (red), medium (blue), and high (green) friction corresponding to 0.5, 1.0, and 1.5 times the standard values used for cell body- and podium-substrate friction (Table 1). The observed faster expansion for lower friction has almost no effect on the number of cells. Shown are mean values of 10 randomly seeded simulations. (TIF) [file pone.0021960.s005.tif]

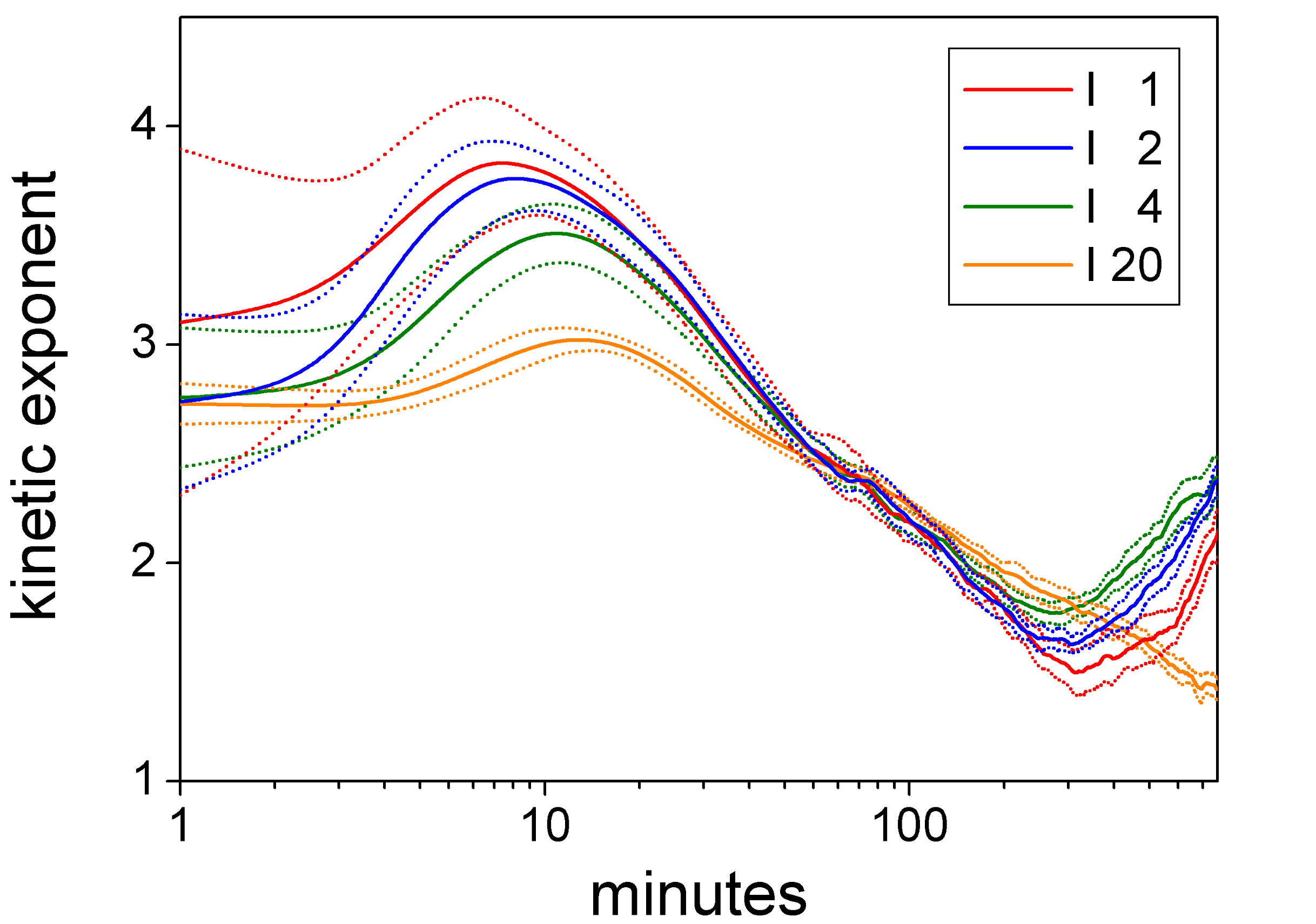

Supplement: Figure S6 — Kinetic exponent for the mean squared displacement (msd) of single non-interacting cells over time for different probabilistic podium inactivation rates. The kinetic exponent characterizes the evolution of the msd with respect to time , i.e. msd . For pure diffusion , for movement with a constant velocity , and for movement with a constant acceleration . For model cells acceleration (force) is proportional to podium length which itself is a function of time. This can result in kinetic coefficients for individual cells. The figure shows mean values (solid) standard deviation (dotted) across 10 simulations running 12000 cells each. The curves vary with respect to the independent podium update rate (red), 2 (blue), 4 (green), and 20/d (orange) that governs probabilistic podium inactivation and thus podium turnover. The curves generally show the same characteristics as the measurements of Dieterich et al. [40] (their Figure 1). Nevertheless, our results show higher values for the kinetic exponent and may thus overestimate ballistic cell movement and acceleration. In addition, the final increase of the kinetic exponent, only seen in the model cells for low and medium podium update rates, is an artifact of our model which is due to cells with trailing podia that move with constant velocity (Modeling methods section E). However, we checked that this effect is without consequences for the results of the main manuscript since cell-cell interactions (not accounted for in this figure) dramatically limit the cell travelling time and range. (TIF) [file pone.0021960.s006.tif]

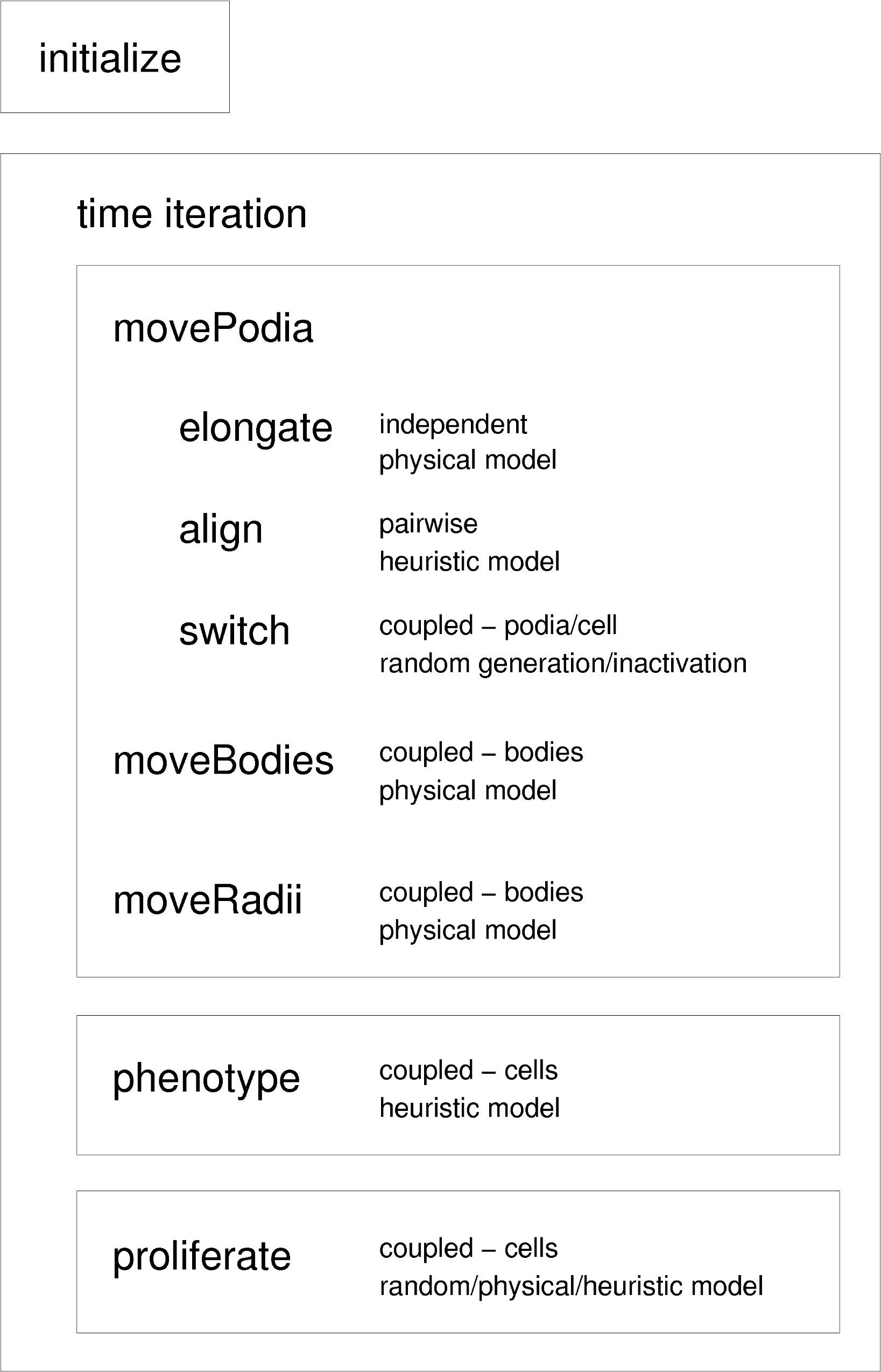

Supplement: Figure S7 — Program flow chart. In each time iteration step, first podia are moved, second bodies, and third radii. Finally, phenotype and proliferation states are updated. The underlying physical, heuristic, and random models are described in the methods section of the main manuscript. Each podium is elongated independently according to the physical model. Overlapping podia are aligned to each other according to the assigned pairwise heuristic. Podia are switched on or off according to random generation and inactivation rates, respectively, which depend on all podia of a given cell. Phenotype (number of podia offset value, independent podia inactivation rate) and proliferation (cell cycle phase, volume growth rate) states are regulated according to the local cell density (accounting for cell bodies and podia). (TIF) [file pone.0021960.s007.tif]
